# Real-time magnetic actuation of DNA nanodevices via modular integration with stiff micro-levers

**DOI:** 10.1038/s41467-018-03601-5

**Published:** 2018-04-13

**Authors:** Stephanie Lauback, Kara R. Mattioli, Alexander E. Marras, Maxim Armstrong, Thomas P. Rudibaugh, Ratnasingham Sooryakumar, Carlos E. Castro

**Affiliations:** 1 Department of Physics, 191 W. Woodruff Ave, The Ohio State University, Columbus, OH 43210 USA; 2 Department of Mechanical & Aerospace Engineering, 201 W. 19th Ave, The Ohio State University, Columbus, OH 43210 USA; 3 Department of Chemical & Biomolecular Engineering, 151 W. Woodruff Ave, The Ohio State University, Columbus, OH 43210 USA; 4 Biophysics Graduate Program, The Ohio State University, Columbus, OH 43210 USA; 5 Present Address: Department of Physics and Engineering Physics, 1700 Moore St., Juniata College, Huntingdon, PA 16652 USA; 6 Department of Physics, 450 Church St., University of Michigan, Ann Arbor, MI 48109 USA; 7 Present Address: Institute for Molecular Engineering, 5640 S. Ellis Ave., University of Chicago, Chicago, IL 60637 USA; 8 Present Address: Department of Bioengineering, 648 Stanley Hall MC 1762, University of California, Berkeley, CA 94720 USA; 9 Present Address: Department of Chemical and Biomolecular Engineering, 911 Partners Way, North Carolina State University, Raleigh, NC 27606 USA

## Abstract

DNA nanotechnology has enabled complex nanodevices, but the ability to directly manipulate systems with fast response times remains a key challenge. Current methods of actuation are relatively slow and only direct devices into one or two target configurations. Here we report an approach to control DNA origami assemblies via externally applied magnetic fields using a low-cost platform that enables actuation into many distinct configurations with sub-second response times. The nanodevices in these assemblies are manipulated via mechanically stiff micron-scale lever arms, which rigidly couple movement of a micron size magnetic bead to reconfiguration of the nanodevice while also enabling direct visualization of the conformation. We demonstrate control of three assemblies—a rod, rotor, and hinge—at frequencies up to several Hz and the ability to actuate into many conformations. This level of spatiotemporal control over DNA devices can serve as a foundation for real-time manipulation of molecular and atomic systems.

## Abstract (web summary)

DNA molecular machines hold promise for biological nanotechnology, but how to actuate them in a fast and programmable manner remains challenging. Here, Lauback et al. demonstrate direct manipulation of DNA origami assemblies via a micrometer-long stiff mechanical lever controlled by a magnetic field.

## Introduction

The ability to control molecular devices in real-time with well-defined temporal and spatial control is a central goal of nanotechnology. Tremendous advances have been made in the assembly of complex nanodevices from DNA^1–4^, amino acid components^5–7^, colloids^8–10^, and nanomaterials^11–13^. In particular, DNA origami nanotechnology^14–16^ has enabled dynamic nanodevices that exhibit complex motion^17–19^, programmed conformational changes^19–21^, long-range motion^22^, and tunable mechanical response^23^, making this a highly attractive approach for the development of nanomachines. The ability to control these nanodevices in real-time is a key step to enable functional robotic systems at the molecular scale. Current methods to actuate DNA nanodevices typically rely on introducing strands that bind to or displace components on the structure to reconfigure a device with response times of ~1 min or greater^18^. Other recent developments have introduced changing buffer conditions such as light or ion concentrations to reconfigure structures^24^, and recent studies demonstrated actuation times on the scale of ~10 s via temperature or pH changes^19,25^. These actuation approaches generally release or facilitate local interactions, and hence control is limited to stabilizing one or two pre-programmed states as opposed to directly and continuously manipulating the device into a specific configuration with an applied force. In addition, one recent study demonstrated a system where local conformational changes triggered by DNA binding are propagated throughout a structure^26^. Although this system passes through several intermediate states as local conformational changes are propagated, it was not possible to directly manipulate the system into these several intermediate states. The goal of this work is to establish a robust approach for the direct real-time manipulation of DNA nanodevices with precise spatial resolution, sub-second response times, and tunable applied forces.

While direct manipulation is challenging at the molecular scale, mechanical control of micro-scale systems is well-established, for example, through manipulation of micron-sized magnetic particles via externally applied magnetic fields^27–30^. The challenge of translating this approach to directly manipulate molecular scale devices is that scaling magnetic particles down in size results in increased thermal fluctuations and decreased forces. For example, Xu et al.^31^ measured forces of <1 femtoNewton for superparamagnetic nanoparticles with a diameter of 30 nm at magnetic fields up to 300 Oe. Previous studies have shown the forces and torques required to reconfigure dynamic DNA origami nanodevices to be on the scale of 1 picoNewton or 10–50 pN∙nm^18^, respectively, which would require superparamagnetic beads at least 1 μm in diameter. Therefore, achieving appropriate actuation forces presents the challenge of a large mismatch in length scales between the actuator and the machine.

In this study, the challenge of bridging microscale manipulation to nanoscale devices is overcome by linking a stiff micro-scale mechanical lever to the DNA origami nanodevices to make micro-scale actuated assemblies. The mechanical lever has a high aspect ratio, where the cross-sectional dimensions are on the scale of the nanomachines (~25 nm), but the length is on the scale of the actuator (~1 μm). To effectively couple the motion of the bead to the nanomachine a highly stiff lever is designed, which allows nearly rigid mechanical coupling of microscale bead motion to nanoscale reconfiguration of the DNA device over lever lengths of at least 1–10 μm. We demonstrate magnetic manipulation of two prototype DNA origami nanomachines, including a rotor system that can exhibit continuous rotational motion, and a hinge system that exhibits a finite range of angular motion. Our approach allows specific control over the angular conformation with resolution of ±8°, continuous rotational motion up to 2 Hz, and the capability of applying up to 80 pN∙nm of torque.

## Results

### Design and fabrication of nanoscale components

**Fig. 1:**
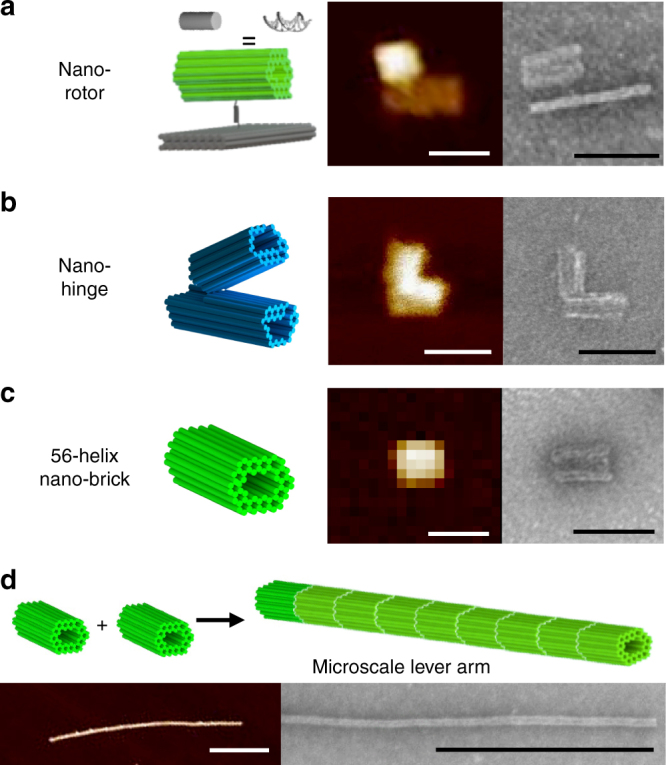
Components for actuated assemblies. The prototype nanomachines include a **a** nano-rotor composed of two separate constructs, the nano-brick and a nano platform, which are connected together via single-stranded DNA (ssDNA) overhang and **b** nano-hinge consisting of two stiff nano-rods with 36 double-stranded DNA (dsDNA) helices joined at one end by 8 ssDNA strand connections. **c** A 56-helix nano-brick composed of 56 dsDNA helices bundled together was used as a basis for a lever arm. **d** The DNA mechanical lever arm for actuation is a 1D array of nano-bricks. Cylindrical models are shown for each with each cylinder representing a DNA helix. AFM and TEM images are shown with scale bar 50 nm for images **a**–**c** and 500 nm for image **d**

Two prototype nanomachines were used to demonstrate our manipulation capabilities, a nano-rotor and a nano-hinge, which are similar to previously reported DNA origami devices^18,32–34^. The nano-rotor (Fig. 1a), our continuous rotational motion prototype machine, utilizes a rotor anchored to a platform by a rotationally flexible joint, so steric interactions facilitate in-plane rotations. The nano-rotor consists of three components: a base platform (schematics and sample images shown in Supplementary Fig. 1; design depicted in Supplementary Fig. 2, and list of DNA staple sequences is provided in Supplementary Table 1), a 56 helix rotor arm (schematics and sample images shown in Supplementary Fig. 3; design depicted in Supplementary Fig. 4, and list of DNA staple sequences is provided in Supplementary Table 2), and a flexible pivot that connects the rotor to the base platform. The nano-hinge (Fig. 1b), our finite angular motion prototype machine, builds on previously published hinge designs^18,33,34^ with two arms connected along an edge via single-stranded DNA (ssDNA) linkers that enable relative rotation of the arms over a finite range of angles. Specifically, the nano-hinge designed in this study is constructed from two ~30 nm long arms, each containing 36 double-stranded DNA (dsDNA) helices, which are connected at one edge by eight ssDNA linker connections. One arm contains an extension on the back side to prevent opening to angles close to 180^o^. Schematics and sample images of the nano-hinge are shown in Supplementary Fig. 5; the design is depicted in Supplementary Fig. 6; and the list of DNA staple sequences is provided in Supplementary Table 3).

For actuation of these two prototype machines, a stiff mechanical lever was designed with the following primary criteria: the length of the lever arm should be ≳1 μm for compatibility with micron beads, and the lever arm should be mechanically stiff to enable effective coupling of bead motion to DNA nanomachines. Therefore, a large cross-section composed of 56 dsDNA helices bundled together to form a nano-brick with a large bending stiffness was chosen (Fig. 1c and Supplementary Fig. 3). This nano-brick was connected end-to-end to construct the mechanical lever (Fig. 1d). For both the nano-rotor and nano-hinge, the cross-section of the rotor or hinge arm components was specifically designed to enable connection to the lever arm.

**Fig. 2:**
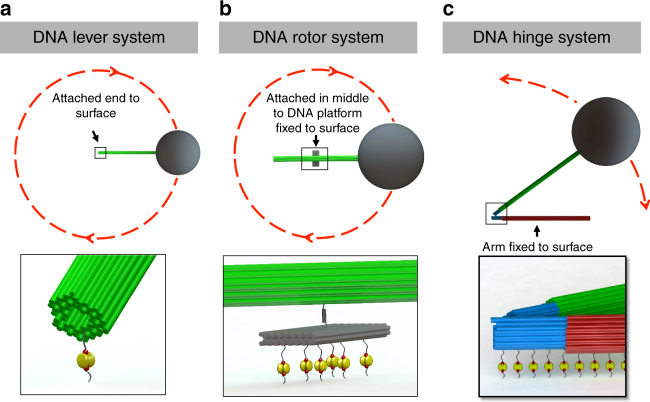
Schematic illustrations of three DNA microsystems. **a** DNA Lever System, **b** DNA Rotor System, and **c** DNA Hinge System—were assembled from DNA nanostructures to actuate three DNA nano constructs—56 Helix nano-brick, nano-rotor and nano-hinge. **a** The nano-brick was attached to the surface via biotin-streptavidin affinity while a micro-lever arm attached to the other end. **b** The nano-platform in the nano-rotor was attached to the surface via biotin-streptavidin affinity, while two micro-lever arms were attached on both sides of the nano-rotor arm. **c** Two micro-lever arms are attached to the nano-hinge. The entire bottom arm of the hinge is fixed to the surface via biotin-streptavidin affinity, while the top micro-arm is free to fluctuate. Micromagnetic beads are attached to the free end of the micro-lever arm in each system. Rotating in-plane fields apply a torque on the bead, precessing the nano-rod and nano-rotor, and opening and closing the nano-hinge

Three assemblies were developed for micro-scale actuation (Fig. 2). First, the mechanical lever was directly actuated as a test system to quantify our manipulation capabilities in terms of position resolution, speed, and force. The mechanical lever is actuated by affixing one end to the substrate and the other end to a superparamagnetic bead. Rotation of the lever arm can then be driven by a rotating magnetic field (Fig. 2a). In the case of the nano-rotor, two stiff levers are coupled to each end of the 56-helix rotor arm to form a micro-rotor. A superparamagnetic bead is added to one of the free ends of the extended lever arm to enable rotation of the nano-rotor about the central pivot between the rotor-arm and nano-platform (Fig. 2b). Similarly, in the nano-hinge system, mechanical levers are connected to both of the nano-hinge arms to form a micro-hinge. One lever arm is fixed to the surface while the other is rotated by a magnetic bead attached to its end (Fig. 2c).

### Assembly/fabrication of micron-scale systems for actuation

**Fig. 3:**
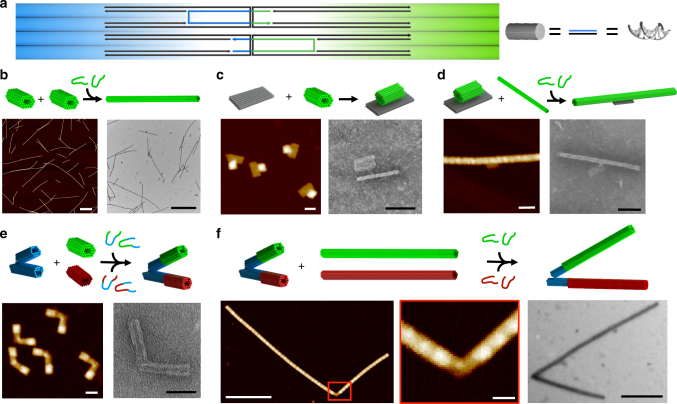
Assembly of systems. **a** ssDNA connecting two structures (polymerization strands) were designed with a u-shaped motif where half have a higher affinity to attach to one side of the interface while the other half have higher affinity to the other side of the interface. **b** Stiff micro levers are assembled by attaching 56 helix nano-bricks end-to-end using polymerization strands. AFM and TEM images show micro-levers. Scale bar is 1 μm. **c** The nano-rotor is assembled by attaching a nano-platform to a nano-brick via a single ssDNA overhang. AFM and TEM images show the nano-rotor construct. Scale bar is 50 nm. **d** Stiff micro-levers are formed off the arm of the nano-rotor using polymerization stands to connect the nano-arm to micro levers. AFM and TEM images show the assembled micro-rotor. Scale bar is 100 nm. **e** A single nano-brick is attached initially to the top and bottom of the nano-hinge using two separate sets of polymerization strands for top (green-blue) and bottom (red-blue). AFM and TEM images show the hinge with top and bottom nano-bricks attached. Scale bar is 50 nm. **f** Stiff micro-levers are formed off the initial nano-bricks by attaching top nano-rods (green) and bottom nano-rods (red) using two separate sets of polymerization staples for the top (green) and the bottom (red). Zoomed out image of AFM and TEM image show a polymerized nano-hinge (scale bars are 500 nm, left and right). Zoomed in image of the nano-hinge from in the AFM image (scale bar is 50 nm, middle)

The microscale mechanical lever arm was constructed from a 1D array of nano-bricks connected via ssDNA strands, which connected the right edge of one nano-brick to the left edge of another (Fig. 3a). These ssDNA strands are referred to as polymerization strands. The lever arm was constructed in a channel by incubating purified DNA nano-bricks with polymerization strands (Supplementary Table 4) at a concentration that was five times in excess of the structure concentration, yielding stiff DNA origami lever arms ranging in length from 1–5 µm (Fig. 3b). The persistence length of the lever was characterized through analysis of shape fluctuations in TEM images, as previously done for actin filaments^35^, as well as by tracking the bending fluctuations of fluorescently labeled levers confined between two coverslips (Supplementary Movie 1), as previously done for actin filaments, microtubules, and amyloid fibers^36,37^. Additional details on the analysis and sample images for both approaches are provided in Supplementary Fig. 7. These methods revealed persistence lengths of 22 ± 4 μm (mean ± standard deviation) and 50 ± 30 μm, respectively. While the methods are in reasonable agreement, the discrepancy may be due to surface deposition influencing the conformation of filaments.

The rotor was assembled by first connecting the platform to the rotor arm (nano-brick) via a single base-pairing interaction flanked by two ssDNA bases on either side for rotational flexibility (Fig. 3c). The nano-brick attached to the platform with an efficiency of ~14% as quantified by gel intensity analysis (Supplementary Fig. 8). Well-formed nano-rotors were purified via gel electrophoresis. Pre-assembled lever arms formed in a tube were then attached to the nano-rotor by incubating the lever arms with the nano-rotors and polymerization strands that connect the nano-rotor to the lever arms (Fig. 3d).

The final system, the extended hinge, was assembled by adding two levers to the nano-hinge to extend each arm to micron-scale lengths. The lever attachment was carried out in two steps. First, a single nano-brick was attached to the top and bottom arms of the nano-hinge (Fig. 3e). To allow specific attachment of a nano-brick to each arm, we designed a different version of the nano-brick that has the same design, but distinct sequences available for binding at the edges of the nano-brick (design depicted in Supplementary Fig. 4). The DNA staple sequences for folding this nano-brick that was attached to the bottom hinge arm are provided in Supplementary Table 5, and the sequences for polymerizing this nano-brick are provided in Supplementary Fig. 6. The specific arm attachment is critical to ensure only one arm attaches to the surface. This approach also decreases the number of steps needed to assemble the extended hinge system by allowing simultaneous attachment of nano-bricks to the top and bottom arms of the hinge in the first step and concurrent attachment of top lever arm (composed of top nano-bricks) and bottom lever arm (composed of bottom nano-bricks). The first nano-bricks were attached to the top and bottom arms of the nano-hinge by mixing them together in equal concentrations with excess polymerization strands (Supplementary Table 7), resulting in 27% attachment of nano-bricks to nano-hinges as quantified by gel intensity analysis (Supplementary Fig. 9). When a single nano-brick is attached to the nano-hinge, the attachment efficiency is 47% (Supplementary Fig. 9). The nano-hinge with one brick attached to each arm was gel purified to remove excess hinge-brick polymerization strands, so they would not interfere with subsequent attachment of the lever arms. The micron-scale lever arm extensions were then added by incubating this assembly with premade micron length rods (Fig. 3f). This approach yielded micro-hinges with typical arm lengths of 1–5 µm.

### Actuation of lever system

**Fig. 4:**
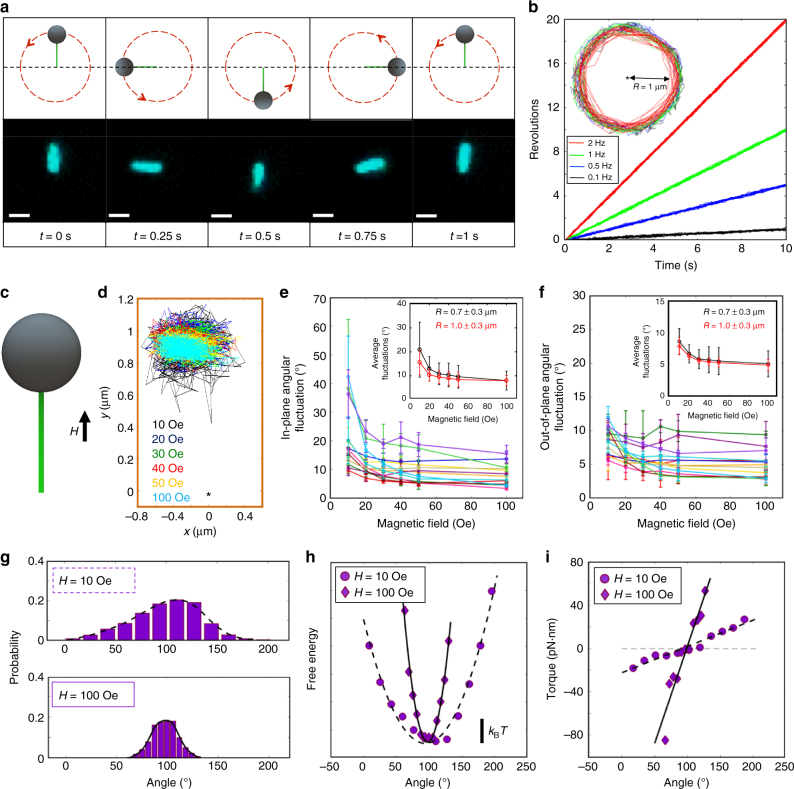
Actuation of micro-lever. **a** Images of the micro-lever rotated over 360 degrees at 1 Hz shown rotating 90° every fourth of a second corresponding to 0, 0.25, 0.5, 0.75, and 1 s. Scale bar is 1 μm. **b** Levers were actuated at four frequencies 0.1, 0.5, 1, and 2 Hz (black, blue, green and red) with rotation traces overlaid for 17 different beads. Inset: Representative tracking of one micro-bead attached to the micro-lever. **c** External in-plane magnetic fields were applied in four orthogonal directions to reorient the lever. **d** Representative tracking of bead fluctuations in an in-plane external magnetic field oriented in the +*y* direction with strengths 10, 20, 30, 40, 50 and 100 Oe (black, blue, green, red, yellow, and cyan). The asterisk indicates the origin. The standard deviation of the **e** in-plane and **f** out-of-plane fluctuations of 13 lever arms, each tested at four orthogonal orientations at every field strength (each color indicates a different lever arm-bead construct, and error bars indicate s.d. over four orientations). Insets show the average and standard deviation of the **e** in-plane and **f** out-of-plane angular fluctuations across all 13 micro-levers (black trace), and the red traces represent the average of the four longest micro-levers. **g** The in-plane angular distribution of the bead shown in purple in **e** and **f** shows greater confinement at 100 Oe (cyan in **d**) compared to 10 Oe (black in **d**). **h** The free energy landscape assuming Boltzmann weighting was calculated from the probability distributions for the same bead at 10 Oe and 100 Oe. **i** The torque on the same magnetic bead was also calculated at 10 Oe (purple circles) and 100 Oe (purple diamonds) by differentiating the free energy landscapes

Each of the three systems was actuated using external magnetic fields provided by four orthogonal electromagnets and a solenoid^28,38^. To quantify our actuation capabilities, the lever arm was directly actuated by attaching one end to a streptavidin functionalized coverslip through a biotin-labeled strand on the structure and the other end to an anti-digoxigenin coated superparamagnetic bead (Dynabeads^®^ MyOne™ Carboxylic Acid) via a digoxigenin labeled strand on the structure. Continuous 360^o^ rotation of the lever was driven by applying a weak (<100 Oe) in-plane precessing magnetic field (Supplementary Movie 2). A torque is applied to superparamagnetic beads due to the anisotropic component of the magnetization, which rotates the bead in a precessing field. Earlier studies characterizing MyOne Dynabeads have shown that in low fields (<30 Oe) a small permanent moment exists^39^, and in high fields (>150 Oe) an anisotropic component of the induced magnetization is manifested as an easy axis such that it is favorable to align according to the local field^40^. Under bright-field, beads attached to tethered lever arms are readily confirmed from their circumferential motion, since detached beads simply spin on their own axes upon actuation. The circumferential motion of the bead rotating about a single point was used as confirmation of a single lever attachment. With our experimental conditions, it was typical to find ~1 lever arm with a magnetic bead attached per imaging field of view (~80 × 80 μm). As illustrated in Fig. 4, it was thus possible to rapidly rotate the lever arm and to tune its speed of actuation up to rotation rates of several Hz with an in-plane precessing field of 40 Oe (Supplementary Movie 2). We further verified that lever arms did not respond directly to a magnetic field when no magnetic bead was attached (Supplementary Movie 3). For magnetic fields (40 Oe) rotating at frequencies beyond 2 Hz, some beads did not consistently track synchronously with the field due to their different magnetic content[40]. The inset in Fig. 4b displays the circumferential path from a representative trajectory of a single bead attached to a lever and actuated at frequencies of 0.1, 0.5, 1 and 2 Hz for 10 s. Levers with lengths ranging from 0.5 to 2 μm were actuated at these frequencies with highly consistent motion. Although the magnetic moments differ from bead to bead^40^, the trajectory vs. time plots of 17 individual beads with varying extension lever lengths, which overlay nearly identically for rotation versus time, demonstrates the reproducibility of each bead rotating synchronously with the field (Fig. 4b).

In addition to driving continuous rotation, it is also possible to hold the lever arm at specific orientations by applying a constant in-plane magnetic field (Fig. 4c). Supplementary Movie 4 illustrates two examples of directly manipulating a lever arm into several different orientations with user-defined control. To quantify our position resolution, 13 lever arm constructs were each restrained at four distinct orthogonal angles under six different magnetic field strengths (10, 20, 30, 40, 50, and 100 Oe). A representative trace of the angular fluctuations of one bead linked to a lever arm constrained to a given orientation is shown in Fig. 4d. For each micro-lever, the in-plane angular fluctuations were measured at four orthogonal locations by tracing the center of the bead in each frame along the circumferential path. Since the lever is highly stiff, radial changes seen in the projections from the bead tracking were interpreted as out-of-plane fluctuations. The standard deviation of the in- and out-of-plane fluctuations for each micro-lever was averaged over the four orthogonal positions, and these measurements were repeated for all thirteen lever-bead constructs at the six field strengths. Figure 4e-f illustrates the in-plane and out-of-plane angular fluctuations for several beads as a function of field strength where each trace represents the behavior of an individual bead-lever construct. The traces show the average angular fluctuations (standard deviation in angle) for the lever arm across four orthogonal orientations, and error bars indicate the variation (standard deviation) of the angular fluctuations across the four orthogonal orientations. The fluctuations in both cases were more confined at higher field strengths. The degree of confinement varied from bead to bead, likely due to the differences of the anisotropic magnetic moment of each bead^40^. The standard deviation of the in-plane angular fluctuations for the 13 lever-bead constructs ranged from ±42° to ±10° at a low field of 10 Oe (Fig. 4e) with out-of-plane fluctuations between ±12° to ±6° (Fig. 4f). However, at larger fields of 100 Oe, the Brownian fluctuations are more suppressed and the standard deviation of in-plane angular fluctuations decreased to lie between ±15° to ±3° with out-of-plane fluctuations between ±9° to ±3°. The inset of Fig. 4e-f show the variability in behavior across all 13 levers (black) and for a subset of 4 longer micro-levers. These results suggest that longer micro-levers may provide improved control and reduced variability between beads, although the differences are only notable at the lowest field strength. However, there is still significant variation across multiple beads even with the subset of longer filaments, suggesting the differences in strength of the magnetic moment and possibly the orientation of the bead relative to the lever also contribute to the variability between beads. On average, including all 13 micro-levers, this approach provides orientation control within ±8^o^ with applied field strengths of 100 Oe, suggesting the constructs can be held in many distinct configurations. This demonstrates a clear advantage over other actuation approaches^18–20^, which are largely limited to switching between one well-defined conformation and another freely fluctuating state.

The extent of the Brownian fluctuations and spread in confinement angles also enables characterization of the strength of magnetic traps at a given field strength. For the bead corresponding to the dataset shown in purple in Fig. 4e-f, the probability distribution was used to calculate the free energy of the trap assuming a Boltzmann probability distribution, and the angular variation of the free energy differentiated to determine the corresponding torque that acts to confine the bead (Fig. 4g-i)^18^. The spread in the probability distribution of the bead held at 10 Oe is greatly reduced when confined at 100 Oe (Fig. 4g). By increasing the magnetic field strength, the trap stiffness, and similarly the torque that acts to confine the bead, can be tuned (Fig. 4h-i).

### Actuation of prototype nanomachines via stiff micro-levers

**Fig. 5:**
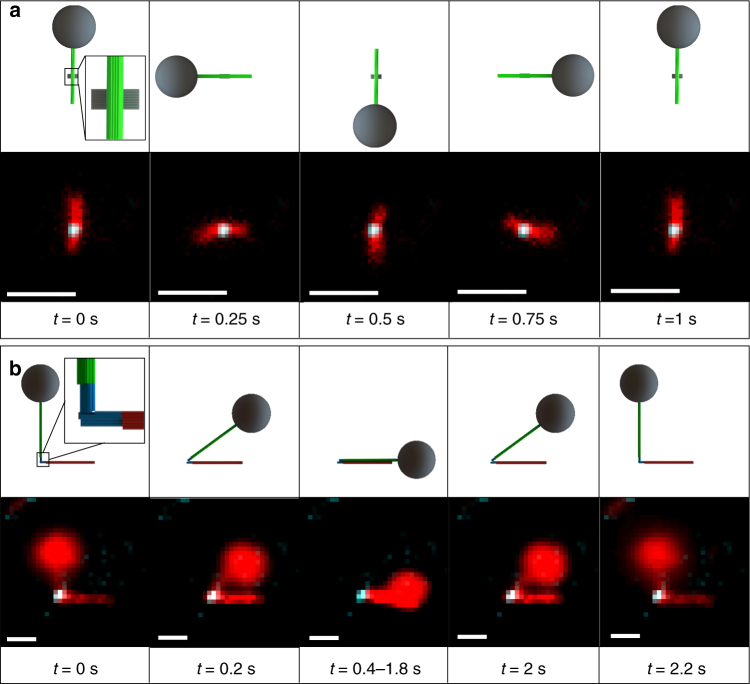
Actuation of prototype nanomachines. **a** Actuation of nano-rotor. Fluorescence images of nano-rotor magnetically actuated in a flow channel via the micro-lever arm attached to micro-magnetic beads. The nano-platform is falsely colored in blue and arms in red with overlapping positions in white. The nano-rotor is rotated by 360 degrees with a frequency of 1 Hz and rotates by 90° every fourth of a second corresponding to video time frames at 0, 0.25, 0.5, 0.75 and 1 s. **b** Actuation of nano-hinge. Fluorescence images of nano-hinge magnetically opened and closed in a flow channel using the extension of micro-lever arms attached to micro-magnetic beads. The nano-hinge is falsely colored in blue and arms in red with overlapping positions in white. Video time shots of the hinge closing (0, 0.2, 0.4 s) and reopening (2, 2.2 s) such that the hinge was left closed from 0.4–1.8 s before being reopened. Scale bars are 1 μm

A similar framework was used to actuate the first prototype nanomachine, the nano-rotor. Micro-rotor assemblies were immobilized to a glass coverslip via biotin-labeled strands attached to the bottom of the platform (Fig. 2b). Superparamagnetic beads were added to the ends of the lever arm via a digoxigenin labeled strand at the end of the lever that binds to an anti-digoxigenin coated bead. By applying a weak (40 Oe) in-plane precessing magnetic field, the rotor pivots about the attachment point to the platform and rotates through 360° as illustrated in Fig. 5a. Supplementary Movies 5 and 6 show two examples of the micro-rotor actuated through continuous rotation at 1 Hz. These results illustrate continuous control over the rotational motion of the micro-rotor. The actuation was repeatable up to 1 Hz. Rotors were actuated up to 2 Hz, but did not track consistently with the rotating field at rotation frequencies higher than 1 Hz (Supplementary Movie 7). This is distinct to the direct actuation of the micro-levers, which could be actuated repeatably at 2 Hz. The different behavior of the micro-rotors may be due to interactions between the lever and platform or enhanced interaction between the lever or bead with the surface.

To enable actuation of the nano-hinge, the bottom lever arm (red arm in Fig. 3f) was attached to a coverslip surface via biotin-labeled ssDNA strands so the opening and closing of the hinge occurred parallel to the plane of the coverslip (Fig. 2c) for easy visualization. The lever arm extension on the mobile arm of the hinge was functionalized with a magnetic bead to allow opening and closing of the hinge by the in-plane external magnetic field (Fig. 5b, two examples are shown in Supplementary Movies 8 and 9). In addition, we demonstrated the ability to hold the hinge at fixed configurations including open, closed, and intermediate angles (Supplementary Movie 10). To estimate the torque required to open or close the hinge, we measured the angular distribution of nano-hinges in the absence of a magnetic field from TEM image analysis. The angular conformations range from ~10° to 165° (Supplementary Fig. 10). A Gaussian distribution fit to the data suggest the equilibrium angle of the hinge is 74° ± 30^o^ (mean ± standard deviation). The free energy was calculated from the probability distribution assuming a Boltzmann distribution (Supplementary Fig. 10) and the torque required to open and close the hinge was calculated as the derivative of the free energy versus angle (Supplementary Fig. 10). These results show that the torque needed to open or close the hinge (e.g., 15 pN∙nm∙rad^−1^) is smaller than the magnetic torque (e.g., 20 pN∙nm∙rad^−1^) supplied by the bead attached to an extended lever arm in a low external field (10 Oe) as shown in Fig. 4i. Therefore, it is possible to actuate the hinge with applied magnetic fields as low as 10 Oe (~20× Earth’s magnetic field). Direct magnetic actuation revealed the hinge can be opened beyond 90° in agreement with the TEM angular distribution (Supplementary Fig. 10).

## Discussion

This work demonstrates control of DNA nanodevices via hierarchical assembly of distinct nanoscale DNA origami constructs into microscale assemblies that can be functionalized with micron-sized magnetic beads for direct manipulation via an externally applied magnetic field. The ability to effectively couple the microscale motion of the bead to the nanoscale reconfiguration of the DNA nanodevice is achieved by designing a highly stiff micron-scale mechanical lever arm. While DNA is generally a flexible polymer, bundling many dsDNA helices into a large cross-section yields a filament with bending stiffness comparable to actin^36^. In order to facilitate attachment to these stiff lever arms, the DNA nanodevices were designed with compatible large cross-sections. In the case of the nano-rotor, the same cross-section was used for the rotating component that attaches to the lever. In the case of the hinge, a smaller cross-section was used that connects to 36 of the helices in the lever cross-section, which resulted in a structurally stable attachment. While even smaller cross-sections could attach to the lever, further reducing the number of helices in the nanodevice components could lead to a weak lever attachment or deformation at the device-lever interface.

Here we focus on driving rotational motion, which allows relatively large movement of micromagnetic beads to be de-amplified to motions on the scale of the nanomachines via the mechanical lever arm. The ability to hold the position of the bead within a standard deviation of ±15^o^ to ±3^o^ at field strengths of 100 Oe suggests our current direct manipulation assemblies could control the position of molecules with resolution as precise as ±2 nm (with ± 3^o^ angular standard deviation) at the ends of the nano-hinge arms, or even more accurately if the molecules were positioned closer to the vertex. This is comparable to the typical position resolution of molecules statically immobilized on DNA origami nanostructures^41^. A prior study demonstrated the ability to position molecules with angstrom-scale resolution using a DNA nanodevice, but this structure was static^34^. Our approach allows direct manipulation of DNA origami nanodevices with nanometer scale precision with sub-second response times and torques ranging from ~20 to 80 pN∙nm∙rad^−1^ at magnetic fields of ~10–100 Oe. The ability to apply tunable torques could enable these platforms to be used for mechanical testing. For example, while the current rotor design contains a highly flexible ssDNA swivel connection, alternative versions could integrate one or more dsDNA helices as the swivel connection to test their torsional response.

We demonstrate continuous rotation of a nano-rotor and oscillating opening and closing of a nano-hinge. Continuous rotation up to 750 radians of rotation (~120 complete rotations) was achieved, which corresponds to 750 μm of bead motion for a 1 μm lever arm. Here we establish continuous rotation of the rotor as the relevant mode of motion. However, based on the quantification of the lever arm control, the rotor could also be held in many distinct conformations. Assuming the average behavior of a micro-lever bead construct (angular control of ±8^o^ illustrated in Fig. 4e), the rotor could be positioned in up to 45 distinct configurations (360^o^/8^o^, i.e., separated by one standard deviation of the fluctuations). Similarly, given an approximate maximum angle of 120^o^ (Supplementary Fig. 10), the micro-hinge could be positioned in up to 15 distinct configurations (120^o^/8^o^, separated by one standard deviation). This illustrates the advantage of direct magnetic manipulation relative to previously established approaches that typically actuate between one fixed and one freely fluctuating state^18,19^ or between a couple stable states^20^. In addition, previous approaches are typically limited to actuation response times of on the scale of minutes or greater^18,21,42^. A couple of recent studies demonstrated actuation response time of ~10 s based on temperature changes^19,25^, and recent work quantified inherent conformational dynamics of DNA origami nanodevices at ~0.1–1 s timescales^43,44^. One prior study integrated DNA origami bundles onto magnetic particles to act as artificial flagella to propel the bead^45^; however, the magnetic actuation was not used to control the origami, rather the origami was used to propel directional motion of the bead. To our knowledge, this is the first demonstration of actuation with direct control over a large range of conformations with sub-second response times.

Given recent advances in DNA origami nanotechnology, the manipulation capabilities established here could be integrated into applications such as controlling enzyme function by bringing an enzyme and co-factor together^46^, probing the conformation and stability of nucleosomes^33^, or to detecting biomolecules in solution via structure closing^47^. In addition, our magnetic actuation approach is amenable to control over more complex devices^18^, which we envision can serve as a foundation for nano- or micro-scale robotic systems based on DNA origami assemblies. Importantly, our manipulation can be carried out using a low-cost platform with off-the-shelf electromagnets costing ~$10 each, meaning the platform could be economically adapted to a wide range of applications. Moreoever, the micro-levers developed here could be integrated with a variety of nanodevices to enable low-cost platforms for real-time manipulation of a wide range of dynamic DNA assemblies. Furthermore, an additional advantage of magnetic actuation is the possibility of actuating multiple magnetic beads in parallel. For example, prior work has demonstrated the ability to guide the motion of several magnetically labeled cells simultaneously^30,38,48^. While the focus of this work was to develop methods for actuating individual nanodevices, the experimental conditions used here resulted in some cases where multiple assemblies could be actuated in the same field of view. Supplementary Movies 3 and 11 show examples of actuating two lever arms and two rotors in parallel, respectively, exhibiting the potential for parallel actuation. However, the constructs may rotate out of phase with our current assembly conditions since the magnetic moment of the beads may be orientated in different directions. Nevertheless, the ability to simultaneously actuate multiple devices opens additional possibility to multiplex the control of molecular interactions or nanomachines, and more complex magnetic manipulation platforms^49^ could be used to overcome the different orientations of magnetic moments of the beads on different assemblies.

## Methods

### Design and fabrication of nanoconstructs

All of the DNA origami nanostructures were designed in caDNAno^50^. The 56 helix bundle (also referred to as the nano-brick) has 56 dsDNA helices connected together in a honeycomb lattice formation creating a cylindrical construct that is about 40 nm in length with a cross-sectional diameter of ~24 nm (Fig. 1c). Schematics and sample TEM and AFM images are shown in Supplementary Fig. 3. The design is shown in Supplementary Fig. 4, and DNA staple sequences are provided in Supplementary Table 2. Several different modification overhangs can be incorporated into the nano-brick to customize its use. A single ssDNA overhang conjugated with biotin is incorporated on the end for surface attachment of the lever arm (Supplementary Fig. 3g). An additional five ssDNA overhangs can be integrated into the side to fix one of the hinge lever arms to the surface (Supplementary Fig. 3h). One ssDNA overhang complementary to a ssDNA overhang on the nano-platform is located on the side of the nano-brick for assembly of the nano-rotor (Supplementary Fig. 3i). Two ssDNA overhangs conjugated with digoxigenin are incorporated on the opposite end of the biotin overhang for bead attachment (Supplementary Fig. 3j). Lastly, 10 ssDNA overhangs that are complementary to ssDNA conjugated with a fluorophore are attached to the structure for visualization under Total Internal Reflection Fluorescence (TIRF).

Most versions of the nano-brick were folded^16^ with 1× FoB (Folding Buffer: 1 mM EDTA, 5 mM NaCl, 5 mM Tris), 18 mM MgCl_2_, 200 nM working stock (oligo solution) and 40 nM 7249 Scaffold. However, the nano-brick with the ssDNA overhang for the nano-rotor fabrication is folded with 22 mM MgCl_2_. Structures were folded in an annealing ramp where the folding reaction was initially heated to 65 °C for 15 min, followed by a constant incubation at 52 °C, 51 °C, and 50 °C for 4 h each and then finally cooled to 4 °C. Two versions of the nano-brick were assembled which have the same structure design (Supplementary Fig. 4), however, the scaffold was shifted by 31 bases in order to create a distinguishable set of polymerization staples for the second version. The polymerization staples for top and bottom nano-brick provided in Supplementary Table 4 and Supplementary Table 6, respectively. Both nano-brick versions were purified via 2% agarose gel electrophoresis^16^ in 0.5× TAE with 4 mM MgCl_2_ (Supplementary Fig. 3k-l). Gel-purified structures were used to polymerize the lever arm. The primary version of the nano-brick (design shown in Supplementary Fig. 4 and staple sequences provided in Supplementary Table 2) was folded with ~70% yield as quantified by gel intensity analysis (representative gel results shown in Supplementary Fig. 3k and Supplementary Fig. 8, lane 4). This analysis compared the intensity of the folded band versus the full intensity of the rest of the lane. Analysis was performed on three separate gel experiments, and yield results did not vary more than ±5%. The second version of the nano-brick (design shown in Supplementary Fig. 4 and staple sequences provided in Supplementary Table 5) folded with approximately 50% yield as quantified by gel intensity analysis (representative gel results shown in Supplementary Fig. 3l and Supplementary Fig. 9, lane 5). The difference in yield is likely due to the differing DNA sequences. Alternative folding protocols could likely increase the yield of the second nano-brick, but we optimized folding for the first version and maintained the same protocols for the second version. Additional representative images of the nano-bricks are provided in Supplementary Fig. 3.

The base nano-platform used in the assembly of the nano-rotor is composed of 32 dsDNA helices arranged into a two-layer rectangular shape with dimensions ~ 60 × 27 × 6 nm. Schematics and sample TEM and AFM images are shown in Supplementary Fig. 1; the design is depicted in Supplementary Fig. 2; and DNA staple sequences are provided in Supplementary Table 1. A single ssDNA overhang was incorporated on the top of the platform for attachment to the nano-brick to form the nano-rotor (Supplementary Fig. 1e), and 22 ssDNA overhangs were included on the bottom that could bind to either a ssDNA conjugated to a Cy3 fluorophore for visualization or biotin protein for surface attachment (Supplementary Fig. 1f). The nano-platform was folded with 1× FoB (Folding Buffer: 1 mM EDTA, 5 mM NaCl, 5 mM Tris), 18 mM MgCl_2_, 200 nM Working Stock, 20 nM 7560 Scaffold (Supplementary Fig. 1g). Structures were folded in a 2.5 half day annealing ramp with initial heating to 65 °C and slow cooling from 65 °C–62 °C at 1 °C/1 h, 61 °C–59 °C at 1 °C/2 h, 58 °C–46 °C at 1 °C/3 h, 45 °C–40 °C at 1 °C/1 h, 39 °C–25 °C at 1 °C/30 min and 24 °C–4 °C at 1 °C/1 min. The structures were gel purified as previously described, and gel intensity analysis revealed a 57% yield of folding (representative gel results shown in Supplementary Fig. 8, lane 5).

The nano-hinge is comprised of two 36hb honeycomb lattice bundles that form the arms, which are connected to each other through 8 ssDNA scaffold connections. Schematics and sample TEM and AFM images are shown in Supplementary Fig. 5; the cadnano design is depicted in Supplementary Fig. 6; and the DNA staple sequences are provided in Supplementary Table 3. On the bottom arm of the hinge, six ssDNA overhangs are incorporated which bind to ssDNA conjugated to biotin for surface attachment. Additionally, eleven ssDNA overhangs which bind to ssDNA conjugated to an Alexa 488 fluorophore were integrated into the structure for visualization under TIRF. The nano-hinge was folded with 1× FoB (Folding Buffer: 1 mM EDTA, 5 mM NaCl, 5 mM Tris), 16 mM MgCl_2_, 100 nM Working Stock and 20 nM 8064 Scaffold (Supplementary Fig. 5e-f). Staples near the ends (Neighbor staples in Supplementary Table 5) of the structures were left out of the folding reaction leaving long scaffold loops which, in turn, reduce base stacking between structures (Supplementary Fig. 5g-l). To fold the hinge, it underwent the same 2.5 day annealing ramp that was used in folding the nano-platform. The nano-hinge was also purified via 2% agarose gel electrophoresis in 0.5× TAE with 4 mM MgCl_2_, and gel intensity analysis revealed a folding yield of approximately 60% (representative gel results shown in Supplementary Fig. 9, lane 6).

### Assembly of extended systems

All of the polymerization strands connecting the nano-bricks to each other and the nano-bricks to the nano-hinge were designed with a u-shaped motif (Fig. 3a). The DNA staple sequences for polymerizing the top nano-brick are provided in Supplementary Table 4. The DNA staple sequences for polymerizing the bottom nano-brick are provided in Supplementary Table 6. The DNA staple sequences for attaching the nano-bricks to the nano-hinge arms are provided in Supplementary Table 7. To form the levers (Fig. 3b), gel purified nano-bricks at ~5 nM concentration were incubated with neighbor and polymerization staples in 5 times excess in an assembly buffer containing 8 mM MgCl_2_ and 0.2% of the surfactant NP40 (to reduce filament aggregation) at a constant temperature of 37 °C for 16–18 h when formed in a channel. When fabricated in a tube, the mixture underwent a thermal ramp starting at 45 °C and decreased by 2 °C every hour until it reached 4 °C for 2 cycles. Additional sample images of micro-levers are provided in Supplementary Fig. 7a-b. This approach yielded micro-levers with lengths ranging from several hundred nanometers up to approximately 5 µm. Manual measurements of lengths from TEM and AFM images (sample images shown in Supplementary Fig. 7a-b) gave an average length of 1.2 ± 0.7 µm. This suggests that on an average ~30 nano-bricks are contained in the micro-levers, given each nano-brick is 40 nm long. Hence, a typical concentration of micro-levers would be ~160 pM (5 nM nano-bricks divided by 30 nano-bricks per lever).

The nano-rotor was formed in an overnight incubation at 37 °C by attaching PEG-purified nano-bricks to PEG-purified platforms via a single ssDNA overhang on each structure that base-pair to connect the side of a nano-brick to the top of the platform (Fig. 3c). Well-formed nano-rotors formed at a yield of 14%, based on gel intensity analysis, and were gel purified for lever arm attachment in the next step (Supplementary Fig 8a, last 4 lanes are repeats). Supplementary Fig. 8b shows a TEM image of gel-purified nano-rotors illustrating nearly all the gel purified structures are properly formed. The lever arms were connected to the gel purified nano-rotors by attaching them to premade micron length levers, which were incubated in 10-fold excess to the nano-rotors and with 5-fold excess polymerization staples relative to nano-brick in a thermal ramp that repeats three cycles of cooling the sample from 45 to 4 °C by decreasing the temperature at a rate of 2 °C every hour (Fig. 3d). Lever arms were attached to nano-rotors at high efficiency, where nearly all the well-formed nano-rotors contained at least one micro-lever. Some free platform structures were observed that were likely leftover from the purification process and a number of free micro-levers were also observed, consistent with the excess micro-levers added. Supplementary Fig. 11 shows a sample TEM images of micro-rotor assemblies.

To form the extended hinge, the initial PEG-purified nano-bricks were attached to the top and bottom arms of the PEG-purified nano-hinge by incubating them together in equal concentrations with polymerization staples that were in 5× excess to the hinge concentration and neighbor staples that were in 2× excess to the hinge concentration (Fig. 3e). This mixture of nano-bricks and hinges was subjected to a thermal ramp starting at 45 °C and decreased by 2 °C every hour until it reached 4 °C. The mixture was then gel purified to extract well-formed nano-hinge-brick constructs that formed with an efficiency of 27% based on gel analysis (representative gel results in Supplementary Fig. 9, lane 9). Gel purification resulted in excellent efficiency of properly formed nano-hinges containing a single nano-brick on each arm as illustrated in a zoomed-out AFM image in Supplementary Fig. 9b. Next, the stiff micro-lever extension arms were connected to each arm of these initial nano-brick-hinge units adding each micro-lever arm, top and bottom, at 2-fold excess, using the same thermal ramp. In this case, the cycle was repeated two additional times since the concentration of the structures was much lower than in attachment of the first unit (Fig. 3f), especially since the two levers (top arm and bottom arm) are at a low concentration. Nearly all hinge-brick constructs contained micro-levers attached, although some nano-hinges (<10%) with only a micro-lever on one arm where observed. In addition, a small number of unbound levers were observed. Micro-hinge assemblies were sparse on TEM images and later in the actuation assays because of the dilution that resulted from combining three components, two of which were the lever arms at low concentration. Some additional sample micro-hinge assemblies are shown in TEM images in Supplementary Fig. 12.

### Surface attachment and bead labeling

To prepare the structures for actuation, each system had to be fixed to the surface in a channel. Nonspecific binding was reduced by initially cleaning the coverslips with piranha solution and coating with unmodified and biotin-modified PEG (10% biotin-PEG) following previously described protocols^51^. 20 μL of free streptavidin (0.1 mg∙mL^−1^) mixed with BSA (0.1 mg∙mL^−1^) was added to the channel and incubated for 5 min to allow for attachment of Streptavidin to the biotin-PEG on the surface. Excess streptavidin and BSA was removed by washing 120 μL of 0.5× TAE with 4 mM MgCl_2_ through the channel, and then biotin-labeled structures were flowed into the channel and attached to the surface. For lever arm actuation experiments, a single nano-brick with a biotin overhang on one end was attached initially to the surface by flowing in 20 μL of 500 nM nano-bricks with the end biotin overhang and incubating at room temperature for 10 min. Excess nano-bricks were removed by washing 120 μL of 0.5× TAE with 4 mM MgCl_2_ through the channel. Subsequently, 20 μL of solution containing 5 nM of nano-bricks without biotin, 25 nM polymerization staples and 0.2% NP40 (NP40S SIGMA TERGITOL™ solution) was introduced into the channel to enable nano-bricks to attach end-to-end. The channels were placed in sealed containers with a reservoir of buffer to prevent evaporation from the channel and incubated for 18–20 h at 37 °C to allow micro-lever polymerization. 20 μL of 1 mg∙mL^−1^ of casein dissolved in 0.5× TAE with 4 mM MgCl_2_ was flowed into the channel to prevent the beads from sticking to the surface and incubated for 10 min. Excess casein was removed by washing 120 μL of 0.5× TAE with 4 mM MgCl_2_ through the channel. Then 20 μL of 0.01–0.1 mg∙mL^−1^ of superparamagnetic beads (Dynabeads® MyOne™ Carboxylic Acid) functionalized with anti-digoxigenin were flown into the channel and incubated for 5 min. Excess beads were removed by washing with 120 μL of 0.5× TAE with 4 mM MgCl_2_ and 60 μL of 0.5× TAE with 4 mM MgCl_2_. This yielded ~3–10 micro-levers properly attached to by one end to the coverslip per imaging field of view (80 × 80 µm) with ~1–3 having properly attached beads. While protocols could have been optimized to improve surface immobilization and bead labeling, we deemed this sufficient for effective characterization of the lever arm manipulation.

For rotor and hinge actuation experiments, the fully extended rotors and hinges were flown into the channel and incubated for 10 min before washing out excess structure with 120 μL of 0.5× TAE with 4 mM MgCl_2_. Finally, superparamagnetic beads (Dynabeads® MyOne™ Carboxylic Acid) functionalized with anti-digoxigenin are attached to the overhangs on the ends of the free lever arms of each structure. To prevent the beads from sticking to the surface, 20 μL of 1 mg∙mL^−1^ of casein dissolved in 0.5× TAE with 4 mM MgCl_2_ was flowed into the channel and incubated for 10 min. Excess casein was removed by washing 120 μL of 0.5× TAE with 4 mM MgCl_2_ through the channel. Then 20 μL of 0.01–0.1 mg∙mL^−1^ of anti-digoxigenin labeled beads were flown into the channel and incubated for 5 min. Excess beads were removed by washing with 120 μL of 0.5× TAE with 4 mM MgCl_2_ and 60 μL of 0.5× TAE with 4 mM MgCl_2_. This yielded similar results for the nano-rotor with ~2–5 properly attached rotors in an imaging field of view with 1–2 rotors containing a magnetic bead. The micro-hinge assemblies were formed at lower concentrations since they contained three components, including two micro-levers, both of which were at lower concentrations. We typically only observed ~1–3 micro-hinges per field of view, and it was unusual to see more than one micro-hinge assembly in a single field of view with a magnetic bead attached.

### Anti-digoxigenin bead functionalization

Carboxyl coated beads (Dynabeads® MyOne™ Carboxylic Acid, Catalog # 65011) were labeled by initially washing and resuspending in 15 mM 2-(Nmorpholino) ethanesulfonic acid (MES) buffer (pH 6.0). Next, the beads were affixed with 1-Ethyl-3-(3dimethylaminopropyl) carbodiimide (EDC) by incubating with 10 mg∙mL^−1^ of EDC for 2 h on a rotisserie followed by a wash step to remove the excess EDC. Then, the beads were incubated on the rotisserie overnight with 2 mg∙mL^−1^ of anti-digoxigenin suspended in 15 mM MES buffer. Last, the excess anti-digoxigenin was removed and resuspended in 0.5× TAE with 4 mM MgCl_2_.

### Persistence length from shape variance in TEM images

TEM images of levers were analyzed using MATLAB (Mathworks, Natick, MA, USA). To discretize the shape of the lever, points along the trajectory were manually selected (~every 50–100 nm along the filament path) by clicking on the image (Supplementary Fig. 7a). These selected points were used to fit a cubic spline of the trajectory coordinates every ~100 nm along the filament path to obtain fine resolution of the curvature. Only filaments that were at least 1 μm in length were considered for the shape fluctuation analysis. Configurational distributions were obtained by aligning the filament trajectories so that they started at the origin and initially pointed in the horizontal direction (Supplementary Fig. 7b). Isambert et al.^35^ derived a relation between the filament persistence length ($L_{\mathrm P}$) and the average transverse fluctuations, ⟨[*D*(*s*)]^2^⟩, or essentially, the splay width for the configurational distributions previously described:

1 $$\left\langle {\left[ {D\left( s \right)} \right]^2} \right\rangle = L_{\mathrm p}^2\left[ {2\frac{s}{{L{}_{\mathrm p}}} + \frac{{16}}{3}{\rm{exp}}\left( { - \frac{s}{{2L_{\mathrm p}}}} \right) - \frac{1}{3}{\rm{exp}}\left( { - \frac{{2s}}{{L_{\mathrm p}}}} \right) - 5} \right].$$

The average transverse fluctuations were determined as a function of arc length from the filament configurational distributions. Only the first 1 μm of all filament trajectories was used for this analysis to avoid averaging transverse fluctuations over a smaller number of filaments for larger arc lengths.

The $L_{\mathrm P}$ of the lever arm was characterized by calculating the average of the transverse fluctuations squared from the configurational distributions and fitting Eq. (1). Supplementary Fig. 7c shows the model fits compared to the data, which resulted in $L_{\mathrm P}$ of 22 ± 4 μm, based on the fit with *R*^2^ = 99.9%. As expected the fluctuations decrease for larger, or equivalently stiffer, cross-sections. The dashed gray lines show the standard error of the mean when calculating the mean of the transverse fluctuations squared as a function of arc length. The uncertainty in the persistence length was determined by fitting Eq. (1) to these dashed gray lines to determine persistence length for transverse fluctuations up to one standard deviation from the mean.

### Persistence length from imaging thermal fluctuations

An alternative method for calculating the persistence length was implemented from measuring the thermal fluctuations from TIRF images of the lever arm sandwiched between two coverslips. Initially, 20 μL of 1 mg∙mL^−1^ of casein dissolved in 0.5× TAE with 4 mM MgCl_2_ were incubated between two coverslips for 10 min followed by a wash with ddH_2_O. Then 0.5 μL of the DNA lever, prepared as previously described and labeled with fluorophores, was sandwiched between the coverslips for imaging. TIRF images were recorded at 5 Hz up to 300 frames. Bending fluctuations were evaluated by calculating an arc length vs. tangent angle trace for each frame. This trace was fit to a Fourier series to determining the bending mode amplitude coefficients, $a_n$, which are related to the persistence length, $L_P$, by the equation:

2 $$\left\langle {\left( {a_n - a_n^0} \right)^2} \right\rangle = L_{\mathrm p}^{ - 1}\left( {\frac{{L_{\mathrm C}}}{{n\pi }}} \right)^2,$$

where $a_n^0$ corresponds to the average shape, $k_{\rm{B}}$ is Boltzmann’s constant, $T$ is absolute temperature, $n$ is an integer corresponding to the bending mode, and *L*_C_ is the contour length. This method was previously used to determine properties of actin filaments, microtubules, and amyloid fibers^36,37^. Results for 10 lever arms give a persistence length of 30 ± 20 μm. Additional details are provided in Supplementary Fig. 7.

### AFM and TEM imaging

Nanostructures were imaged using a Bruker AXS Dimension Icon AFM (Bruker, Billerica, MA). DNA nanostructures were fixed onto a 12 mm Mica Discs (Ted Pella, Inc., Redding, CA). By removing a strip of double-sided tape adhered to the mica substrate, a mono layer of mica was formed. Next, 5 µL of structures at 1 nM were incubated on the mica substrate for 2 min and washed off with 1 mL of ddH_2_O. The substrate was quickly dried using an air gun and filter paper. The structures were imaged on the AFM in Scanasyst Air mode. TEM imaging samples were prepared as previously described^16^. The samples were imaged on a FEI Tecnai G2 Spirit TEM.

### Magnetic manipulation

External magnetic fields were applied using four orthogonal electromagnets, which provided in-plane magnetic fields and a central solenoid which provided out-of-plane magnetic fields. The magnetic setup was designed for an inverted microscope which utilized small electromagnets (1.25 inches long, 1.25 inches in diameter, OP-1212, Magnetech Corp) and for an upright microscope which utilized larger electromagnets (2.5 inches long, 2 inches in diameter, OP-2025, Magnetech Corp).

### Data availability

The nanostructure designs and all relevant data generated during and analyzed during the current study are available from the corresponding authors on reasonable request.

## Electronic supplementary material

MgCl_2_Supplementary Information

Description of Additional Supplementary Files(PDF 177 kb)

Supplementary Movie 1

Supplementary Movie 2

Supplementary Movie 3

Supplementary Movie 4

Supplementary Movie 5

Supplementary Movie 6

Supplementary Movie 7

Supplementary Movie 8

Supplementary Movie 9

Supplementary Movie 10

Supplementary Movie 11
