# Supplementary material for: Real-time magnetic actuation of DNA nanodevices via modular integration with stiff micro-levers
Source: Nat Commun. 2018 Apr 13;9:1446. doi: 10.1038/s41467-018-03601-5 (PMC5899095; doi:10.1038/s41467-018-03601-5)
Supplement: Supplementary file 1 — MgCl2Supplementary Information [file 41467_2018_3601_MOESM1_ESM.pdf]

Supporting Information:

Real-time Magnetic Actuation of DNA Nanodevices via Modular Integration with Stiff Micro-levers

Stephanie Lauback, Kara R. Mattioli, Alexander E. Marras, Maxim Armstrong, Thomas P. Rudibaugh, Ratnasingham Sooryakumar, and Carlos E. Castro

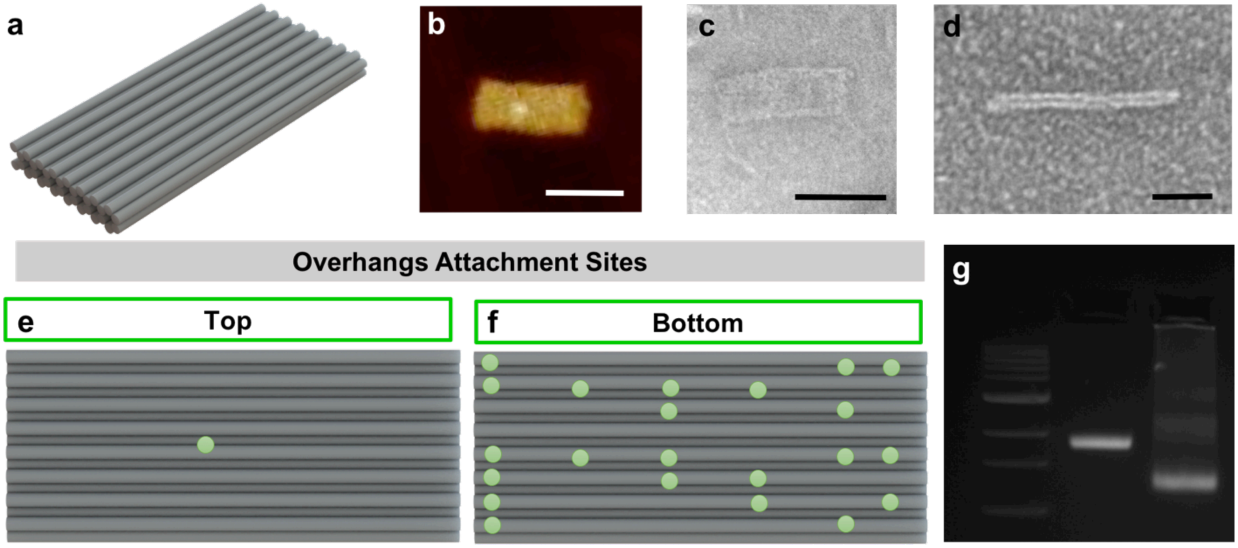

Supplementary Figure 1 Nano-Platform Structure. (a) Cylinder model of the nano-platform. (b) AFM and (c) TEM images of top view of nano-platform. In the AFM image, the nano-platform has a streptavidin molecule attached to top overhang shown in (e) to illustrate the location. Scale bars, 50 nm. (d) TEM image of the side of the nano-platform. Scale bar, 20 nm. Overhang attachment sites for the nano-platform on (e) top (for attachment to nano-rotor) and on (f) bottom (for attachment to coverslip surface or fluorophore attachment). (g) Gel purification of nano-platform at 0.5x TAE with 4mM $MgCl_2$. Gel order: One Kilobase Ladder, 7560 Scaffold, Nano-platform.

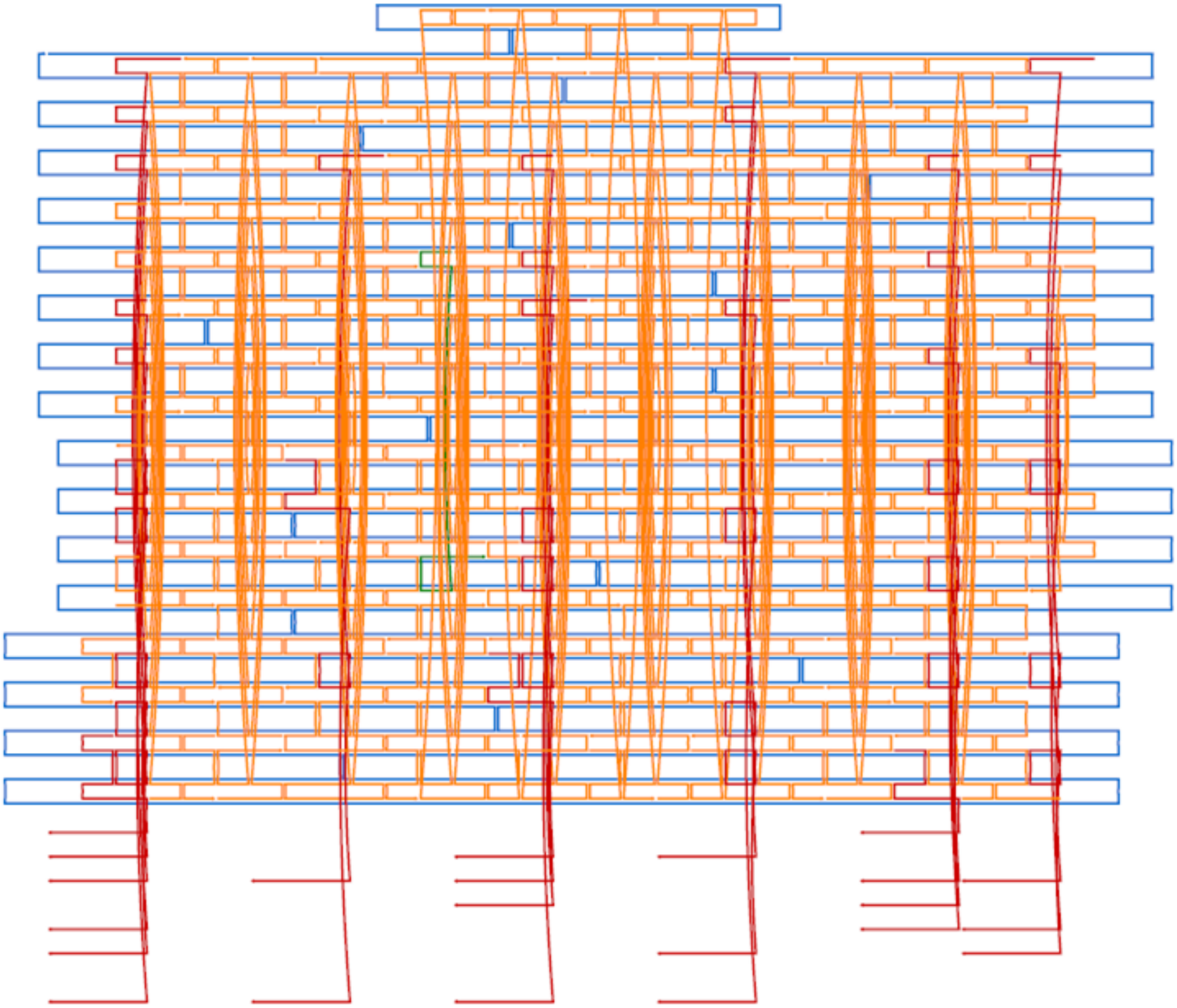

Supplementary Figure 2 CaDNAno design of nano-platform. Scaffold routing is shown in blue. Core staples are shown in orange. Bottom overhangs which attach to ssDNA conjugated with biotin or a fluorophore are shown in red and the overhang on the top which is complementary to an overhang on the nano-brick is shown in green.

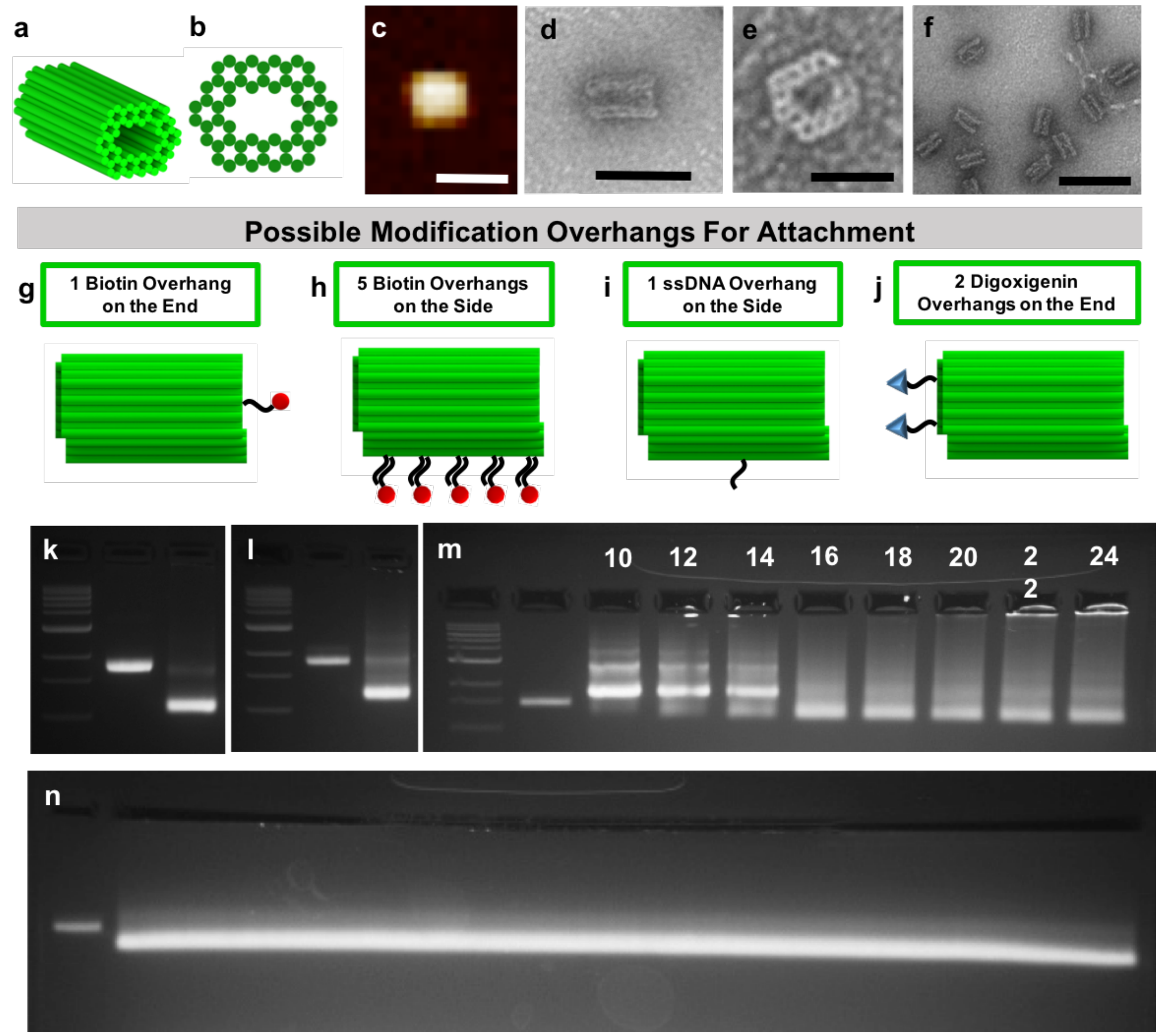

Supplementary Figure 3 Nano-Brick Structure. (a) Cylinder model and (b) cross-section of the 56hb nano-brick. (c) AFM and (d) TEM images of side view of nano-brick. Scale bars, 50 nm. (e) TEM image of the cross-section of the nano-brick. Scale bar, 20 nm. (f) Zoomed out TEM image of gel-purified nano-bricks, scale bar, 100 nm. Modifications can be made to the nano-brick for different attachments including biotin for attachment to the coverslip surface or digoxigenin for attachment to an anti-digoxigenin coated magnetic bead. Nano-brick with (g) one biotin overhang on the end (for lever arm surface attachment in lever actuation studies), (h) five biotin overhangs on the side (for surface attachment of bottom arm micro-lever in nano-hinge actuation studies), (i) one ssDNA overhang on the side (for attachment to nano-platform in forming nano-rotor), (j) two digoxigenin overhangs on the end (for bead attachment used in all actuation studies). The fluorophore overhang attachment sites are not shown. Gel purification of (k) Nano-brick version one and (l) Nano-brick version two (scaffold shift by 30 bases) in 0.5x TAE with 4mM $MgCl_2$. Gel order: One Kilobase DNA Ladder, 7249 Scaffold, Nano-brick. (m) Salt screen of nano-brick version two with $MgCl_2$ concentrations ranging from 10 mM to 24 mM.

Gel Order: One Kilobase DNA Ladder, 7249 Scaffold, Nano-brick Salt Screen (last 8 wells). (n) Purification of nano-brick version one using a large well. Gel Order: 7249 Scaffold, Nano-brick (large well).

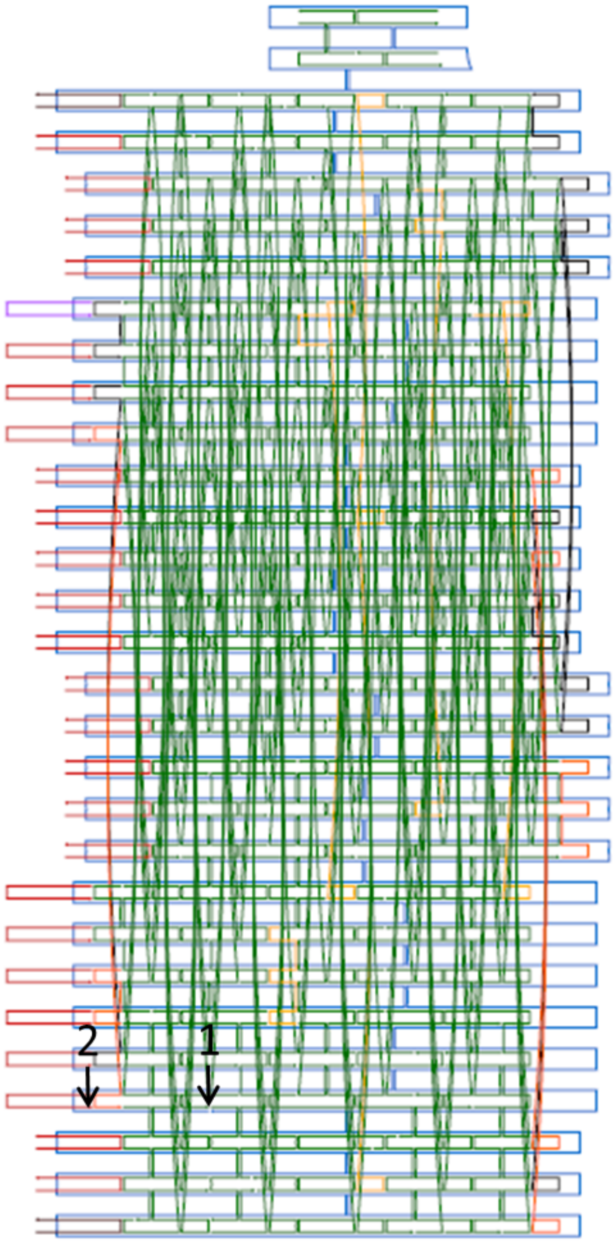

Supplementary Figure 4 CaDNAno design of nano-brick. Scaffold routing is shown in blue. Core staples are shown in green. Neighbor staples are shown in black. Core overhangs for fluorophore attachment are shown in orange. Neighbor overhangs for fluorophore attachment are shown in yellow. Digoxigenin overhangs are shown in brown. Biotin overhang is shown in purple. Polymerization staples are shown in red. The arrows indicate the starting position of the scaffold for nano-brick versions 1 and 2. The scaffold starting point was shifted by 31 bases to the left for version 2.

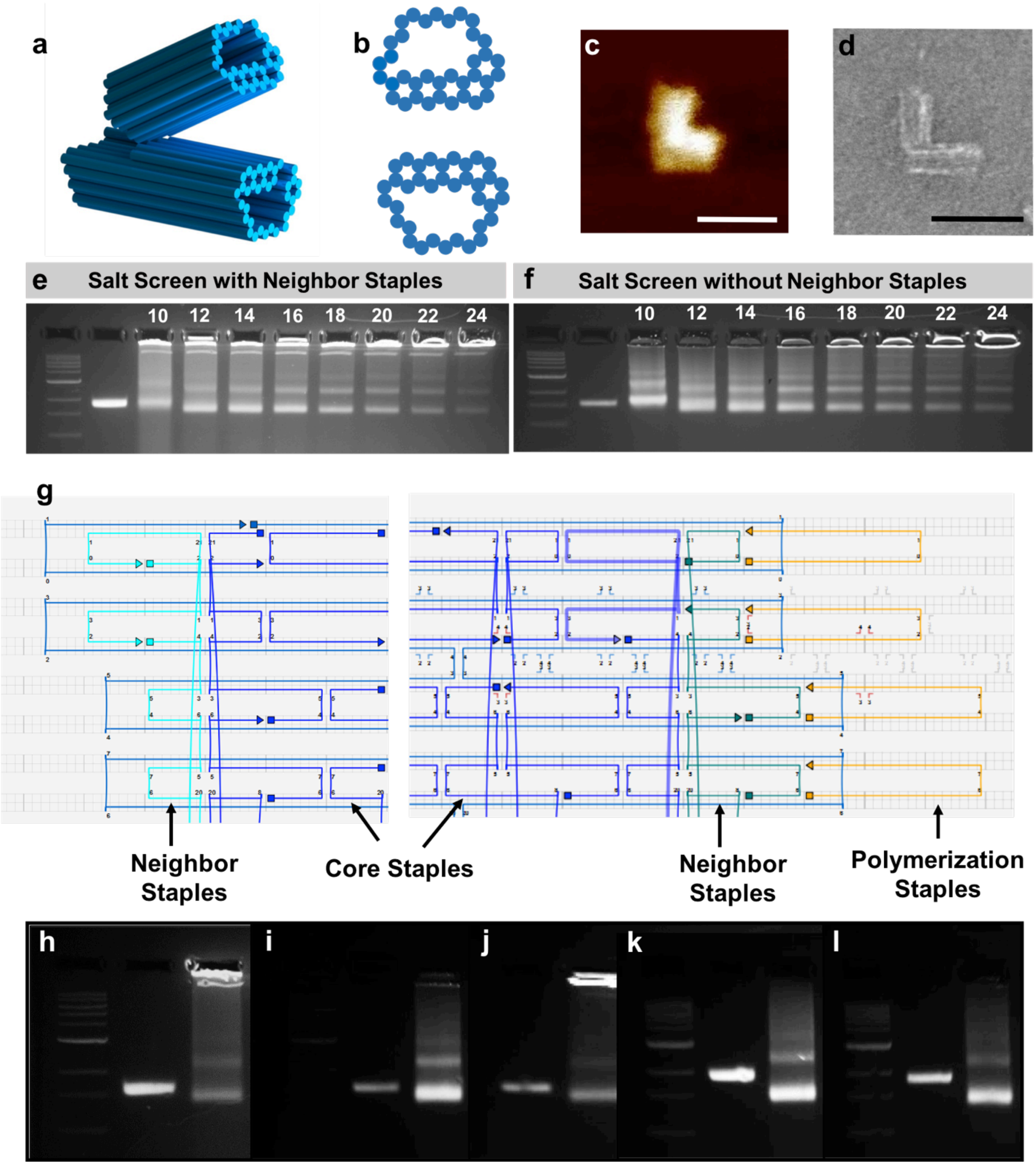

Supplementary Figure 5 Nano-hinge Structure. (a) Cylinder model and (b) cross-section of the nano-hinge. (c) AFM and (d) TEM images of a side view of the nano-hinge. Scale bar, 50 nm. Salt screen of nano-hinge folded (e) with neighbor staples and (f) without neighbor staples over concentrations ranging from 10 mM to 24 mM $MgCl_2$. Gel order for both (e) and (f): One Kilobase DNA Ladder, 8064 Scaffold, Nano-hinge Salt Screen (last 8 wells) Nano-hinge salt-screen. (g) Part of the caDNAno file is depicted to illustrate the Nano-hinge connection sites showing the neighbor staples (cyan), core staples (blue) and polymerization staples (yellow). To

optimize the fabrication, the nano-hinge was folded with different sets of staples and concentrations of scaffold (staples were maintained at 5-fold excess) followed by gel purification. Gel purified nano-hinge (h) at 40 nM scaffold concentration folded with core and neighbor staples, (i) at 40 nM scaffold concentration folded with core staples and immediately purified after folding, (j) at 40nM scaffold concentration and purified a week after folding, (k) at 20 nM scaffold concentration folded with core staples and immediately purified after folding, and (l) at 20 nM scaffold concentration folded with core staples and purified a week after folding.

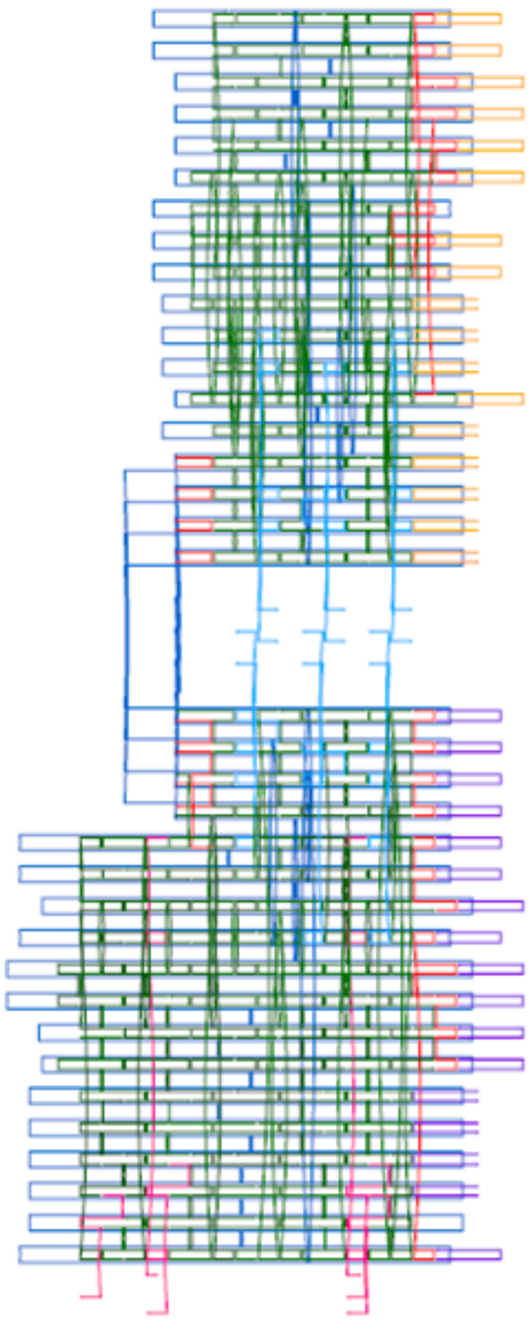

Supplementary Figure 6 CaDNAno design of the nano-hinge. Scaffold routing is shown in blue. Core staples are shown in green. Neighbor staples are shown in red. Overhangs for fluorophore attachment are shown in cyan. Bottom overhangs for biotin attachment are shown in pink. Polymerization staples for attaching nano-brick one are shown in yellow. Polymerization staples for attaching nano-brick two are shown in purple.

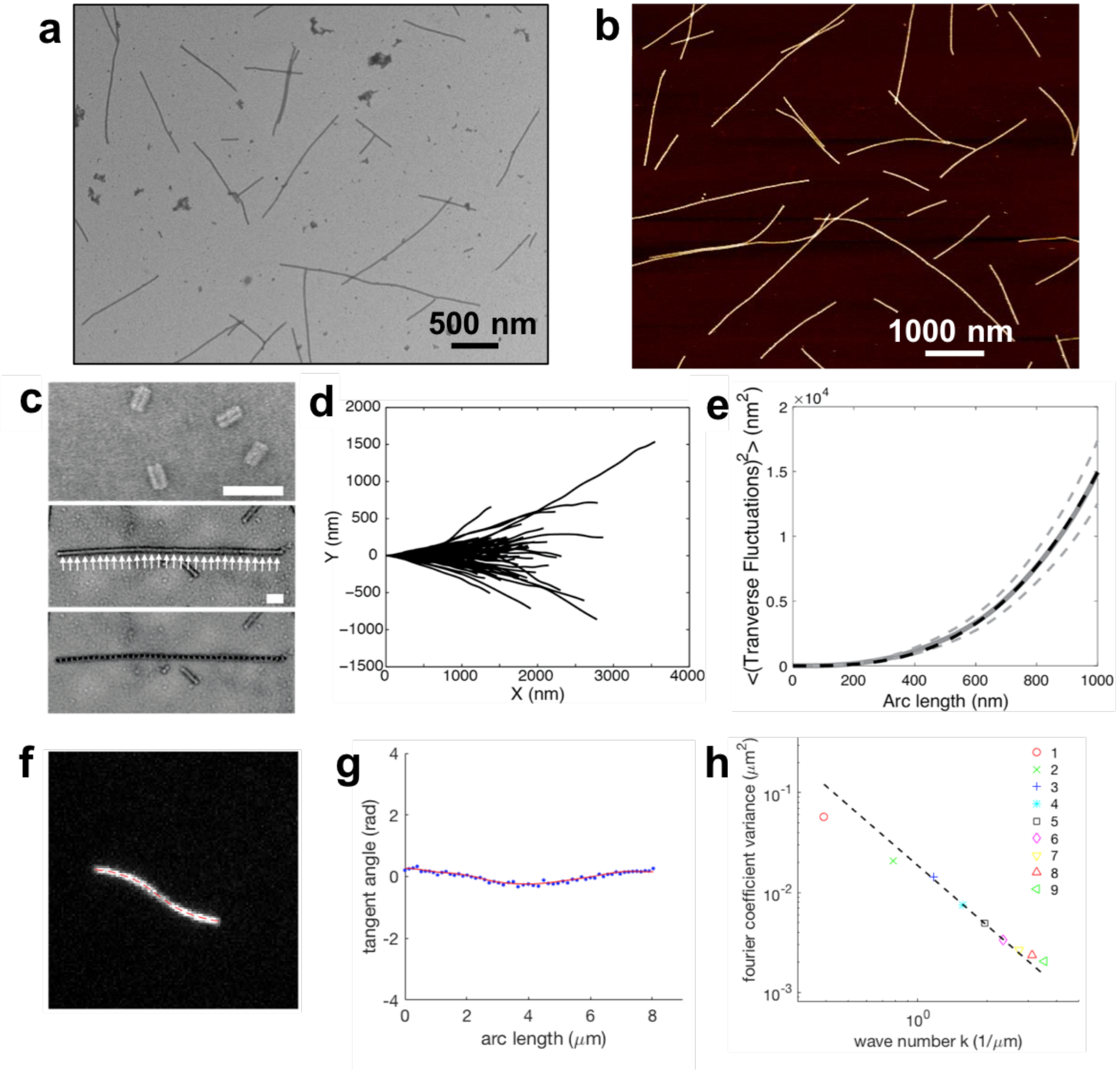

Supplementary Figure 7 Persistence length measurements of micro-lever. Sample (a) TEM and (b) AFM images of micro-levers. (c-e) Shape fluctuation analysis of shape of TEM Images. (c) TEM images illustrating 56-helix DNA origami bundles (top), lever arm filaments constructed from many 56-helix structures as indicated by the arrows (middle), and a manually traced trajectory fit with a cubic spline (bottom). Scale bars, 100 nm. (d) Many lever arm filaments were traced to construct a conformational distribution. (e) The variance of the transverse fluctuations were fit to equation (1) to give a persistence length of 22±4 μm. The dashed lines indicate ± standard deviation of the variance in the transverse fluctuations, which were used to determine the uncertainty in persistence length. (f) Shape fluctuations of individual lever arms were imaged via fluorescence microscopy as shown by a sample image, where the filament is 8.1 μm. (g) The shape of the polymer was decomposed into an arc length versus tangent angle trace (blue circles), which was fit to a fourier series (red line) to determine bending mode coefficients. (h)

The variance of the coefficients was used to determine the filament persistence length as described in equation (2) from the main test. This approach revealed a persistence length of 50 ± 30 μm based on analysis of 13 lever arm filaments, which is in reasonable agreement with results from TEM analysis. The relative difference may be a result of forces during surface deposition causing additional deformations during TEM sample preparation.

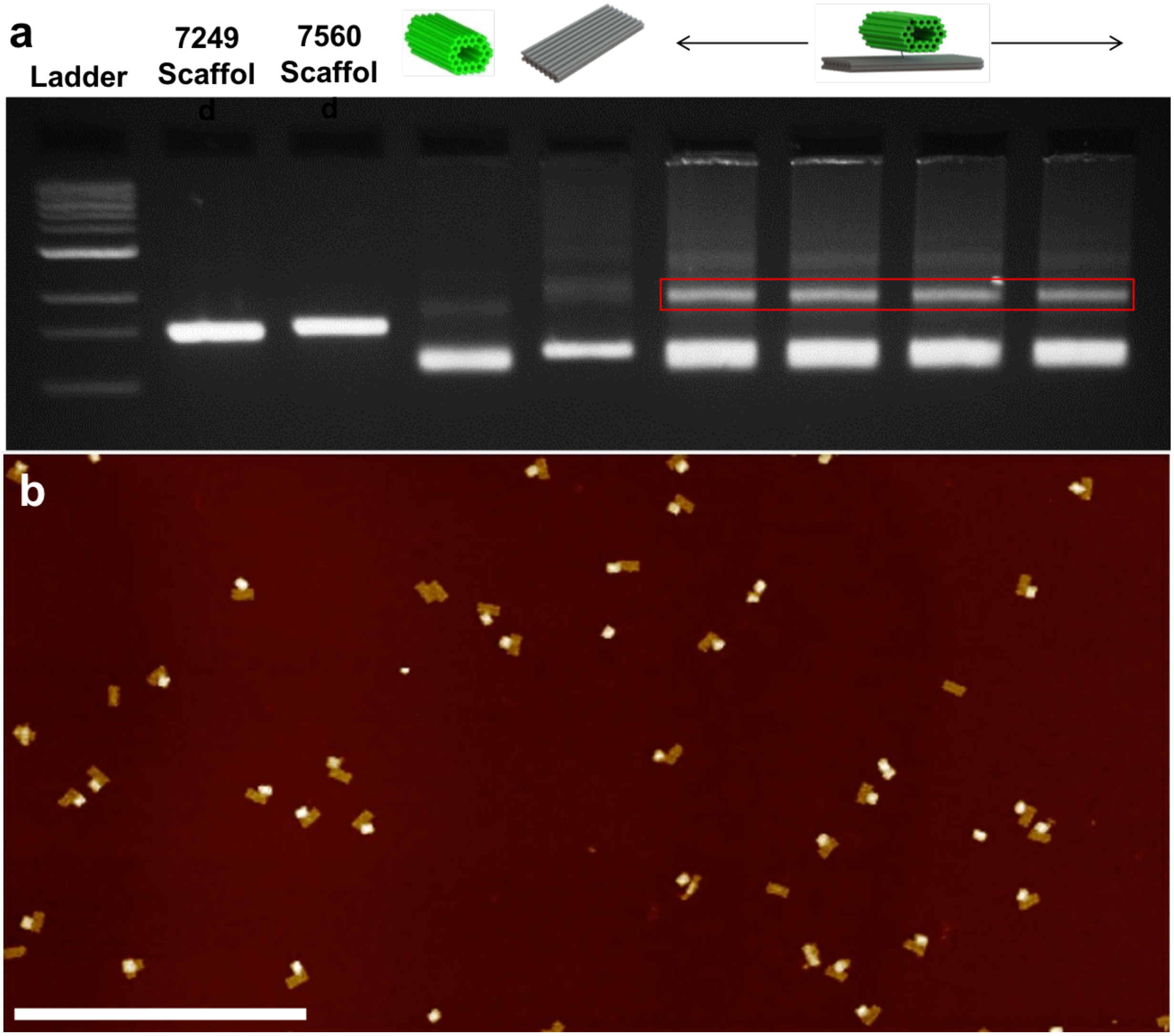

Supplementary Figure 8 Gel purified nano-rotors. (a) Nano-rotors were gel purified. Gel Order: One Kilobase DNA Ladder, 7249 Scaffold (used to fold the nano-brick), 7560 Scaffold (used to fold nano-platform), Nano-bricks, Nano-platforms, and the last 4 lanes are an incubated mixture of nano-bricks and nano-platforms. The lanes corresponding to properly formed nano-rotors are boxed in red. (b) AFM image of nano-rotor band (red box) cut out from gel. Scale bar, 1 µm.

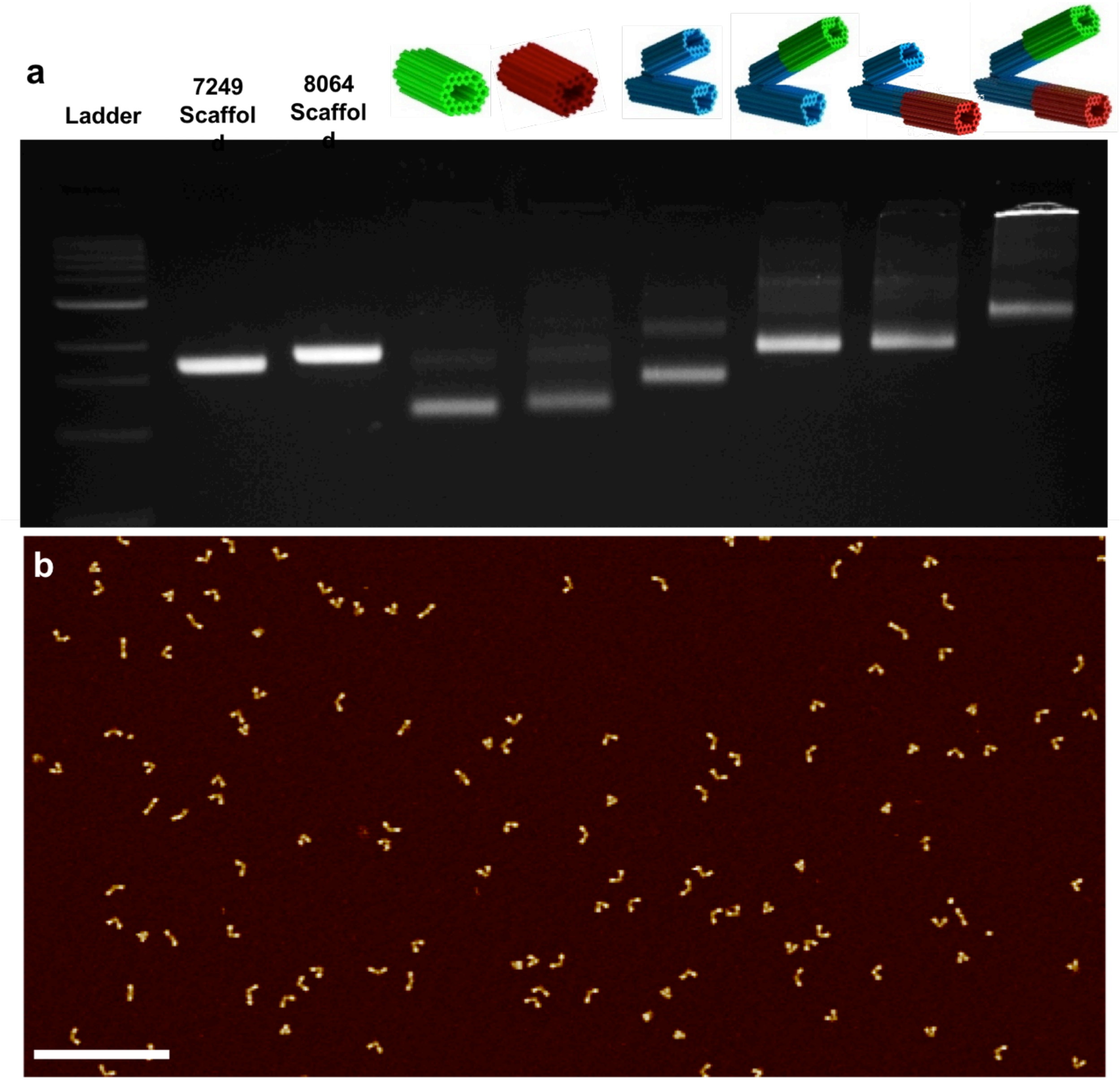

Supplementary Figure 9 Nano-hinge with initial nano-brick attachment. (a) Assembly of nano-bricks to each arm of the hinge was verified via gel. Gel Order: One Kilobase DNA Ladder, 7249 Scaffold (folds the nanobricks (red and green)), 8064 Scaffold (folds the nano-hinge), nano-hinges incubated with green nano-bricks and corresponding polymerization staples, nano-hinges incubated with red nano-bricks corresponding polymerization staples, nano-hinges incubated with green nano-bricks, red nano-bricks, and both sets of corresponding polymerization staples. The nano-hinge with a single nano-brick runs slower than the single nano-hinge or the single nano-brick but faster than the nano-hinge attached to two nano-bricks. As seen in the last three lanes, almost all nano-hinges and nano-bricks attached to one another, although some aggregation is observed in the well. (b) AFM image of gel purified nano-hinge with both nano-bricks attached. Scale bar, 1μm.

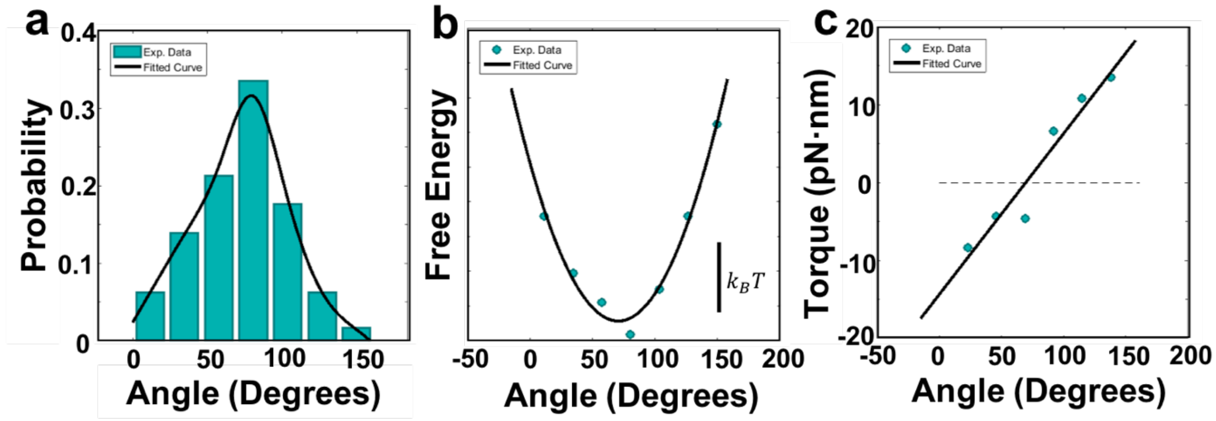

Supplementary Figure 10 (a) Angular distribution of static hinges measured from TEM show a preferred angle of being open at 74˚ with an ability to open to 165˚. (B) The energy landscape assuming Boltzmann weighting was calculated from the probability distributions. (b) The torque required to hold each hinge at a specific angle was calculated by differentiating the from the energy landscape.

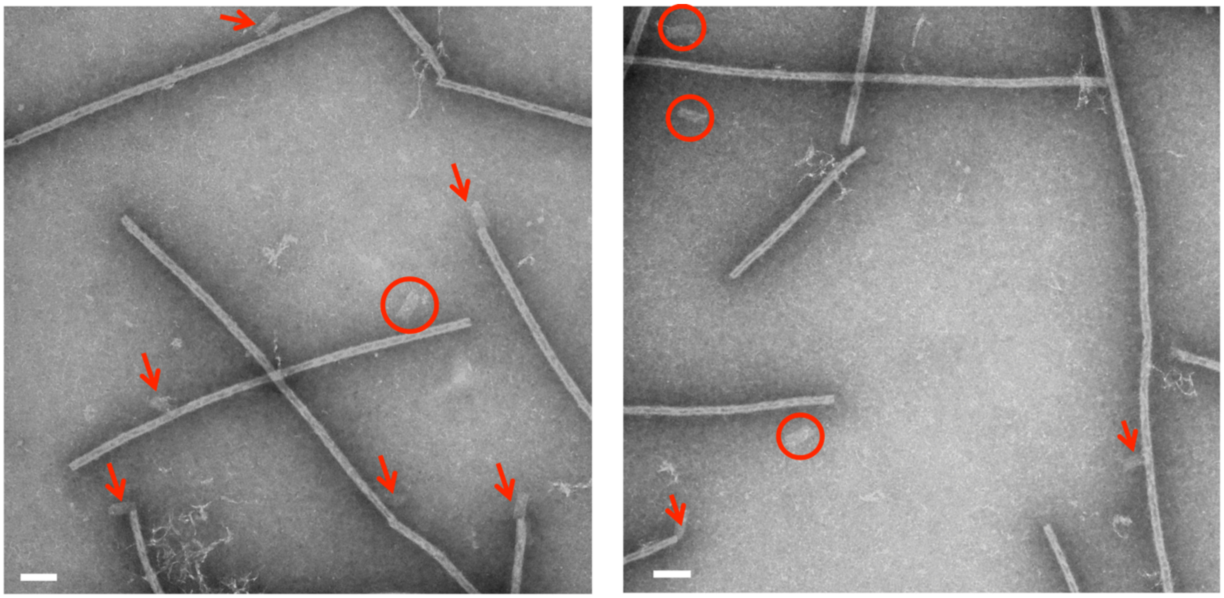

Supplementary Figure 11 Sample TEM images of micro-rotor assemblies containing nano-rotors and micro-lever arms. The platform structures of the nano-rotor are indicated by the red arrows. Free platform structure is indicated by the red circle. Some free (e.g. not attached to nano-rotors) micro-levers were also observed in the fabrication.

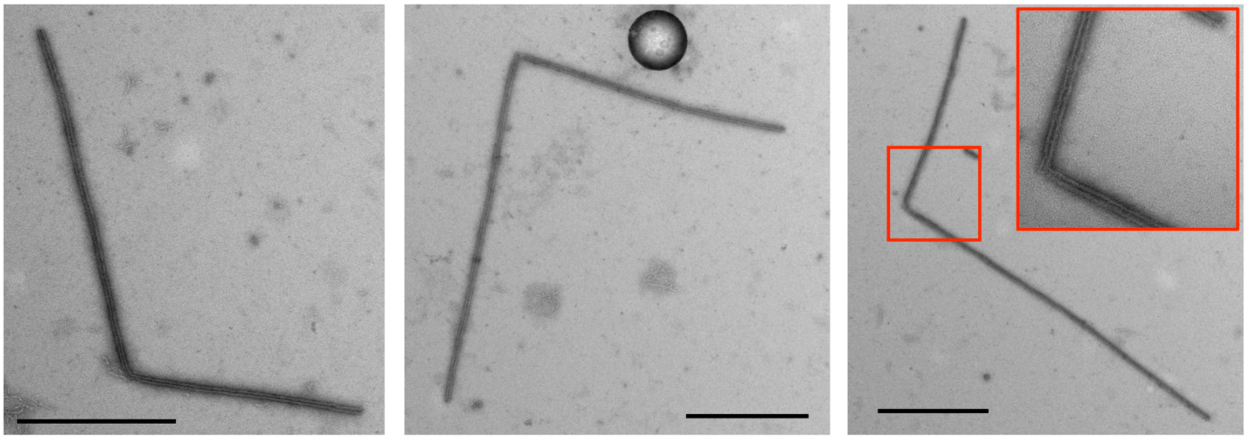

Supplementary Figure 12: Sample TEM images of nano-hinges with micro-levers added to each arm. The efficiency of assembly was high in that nano-hinges not containing micro-levers were rarely observed, but the concentration yield is low for multiple reasons. First, there are additional assembly steps required since individual nano-bricks were first added to the nano-hinge. Second, the concentration of levers is low because we start with a fixed concentration of nano-bricks and allow them to polymerize into long levers. Furthermore, we add two different micro-levers again increasing the number of components and diluting the overall assembly. Scale bars, 500 nm.

Supplementary Table 1. List of ssDNA staple sequences for folding the nano-platform. The start and end columns denote the helix[base] positions at the 5' and 3' ends of the DNA staple strands in the cadnano design (Supplementary Fig. 2). Color is also with respect cadnano.

Start	End	Sequence	Type	Color
39[112]	2[119]	CTTATAAGGTGGTTCCATCGATAGCAGCTCAGACGAGCCCCCGTGGCGA	Core	Orange
24[69]	27[69]	CTCCCGATTACCAAGTTAAGCATAGCCG	Core	Orange
1[98]	27[97]	CTAAATCAAAGCGAAAGGAGCGTGCTTTTATGTTACCGAGGA	Core	Orange
27[56]	6[56]	TAAGCAGCCAATAATAATCAGCCCTTCT	Core	Orange
6[139]	9[146]	ATGGATTGTAACAAAAAGGCTACAACCATCGCCCACGGCTGTAATAGAT	Core	Orange
31[56]	2[56]	CAAAATCCGGAAATTCACCCAGTCACGC	Core	Orange
27[84]	3[104]	AAGGAAAGCAAACGAAAGGGCGACATAATAAATCCTACGCAGCCCACGA	Core	Orange
8[181]	20[182]	ATTCGCGAACTAGCAAAGAGGGCGAAACAAAGTACGCTGGCT	Core	Orange
17[119]	18[105]	ATCGCAAAATCAAAAATCATTTCCCTTACTGATGCTTATACA	Core	Orange
6[244]	22[238]	CCAGCTTTGAGGGGACGACGATGTTAAAATTCGCATGTATAAGAGGAAG	Core	Orange
23[77]	11[76]	AAGAACGAAGCAAGAAATCAAAACATTATCATTTTTGACCCT	Core	Orange
19[105]	24[105]	AGTAAATATTCAGTTAATATCTAAACCAGATACCGATCGGTT	Core	Orange
17[224]	12[231]	AACCCTCTAAAATGACCAGACCGGAAGCGAGGTCACAATTCTAACCTGT	Core	Orange
16[132]	19[132]	GACCATAGACAAAGGCCTGTTTTTAATT	Core	Orange
23[203]	11[202]	CGGGATCGCCACTAGTTGATATCTGGAGCAAACAAAGGCCGG	Core	Orange
29[203]	26[196]	TTTAACGGCTGAGATGATACCGGTAACGCCAGGGTAATATTATGTAGCA	Core	Orange
19[98]	14[98]	AACGAGTAATTCTTGCGTAGAGAGCAAA	Core	Orange
6[97]	26[84]	TAATAAAAAACATCTTGCTTTCGAGGAGTTTCCAGGAGATAA	Core	Orange
13[105]	9[111]	TTTTCAGTAAAGCCTTCAACGGAACGTTGGTTATC	Core	Orange
27[161]	6[161]	CTCAGGACAGGGAACTCAAACCCTACAT	Core	Orange
19[140]	16[133]	TAATCATAATTACTAGTACCTTTACAAAATCGCGCATAGTCACGAGAAT	Core	Orange
23[224]	7[237]	CGAAAGATAAACGGAAAAACAGGAAGATTTAAATTGCCAGTT	Core	Orange
22[125]	19[118]	CCAAGAATCTTTAGTAAATCCTTTGCCCCAAGGATGCTGCTCTGGGCTT	Core	Orange
31[70]	2[77]	GCACCATTACCATTGGGAGGGGGGTCGAAGGGCGC	Core	Orange
2[90]	0[77]	GGGCGCTGGTGCCGAGTCCACTATTAAA	Core	Orange
14[223]	19[216]	GGATGGCAAAGCGATTTAGACTGGATAGAGCAACAAAAAGGAAAATCTA	Core	Orange
39[175]	2[182]	CCCTTCACTGAGAGAGCGCGTTTTCATCTCAGAGCCTGTTTCCAGGGCT	Core	Orange
21[224]	12[224]	CGAAATCTTGAAAGTATGATATTAGCTA	Core	Orange
29[182]	5[181]	AGGAGTGGATTAGGGCTCGCCGTCACGACGTTGTACTTGCTG	Core	Orange
9[98]	20[91]	TGAGGAAATTAATTTTAAAAGGGAGAAGGCTTGCC	Core	Orange
21[231]	25[237]	CGCGACCTTTCCATCAGCATCGCTAAACTCGTCTT	Core	Orange
25[210]	28[210]	GCGTAACCAAACTACCACCCTTTAAGAG	Core	Orange
29[147]	0[140]	TTCCAGTGCCAGCAATCAAGTCTGGTTTGCCCCAGTTCTTTTTGCGTAT	Core	Orange
26[209]	2[210]	CAACGCCCCAAGCGCCATTCGTAAGTTGGACAGTGTGACCTC	Core	Orange
26[181]	8[182]	GAATAACATGAAAATAGCAGCCTAAAGGATGGGATAAAAATA	Core	Orange
25[70]	8[77]	CGCTAACGAGCGTCGCGTTTTAACTGATTGCCACG	Core	Orange
15[147]	10[147]	AGCGGATTACCAAGTTTACATTAAATCAAAATGCAACTTTAC	Core	Orange
31[224]	2[224]	CCATCTTGAGCCGCAACATACATTTCTC	Core	Orange
9[245]	19[244]	GCAAATATTTTGAGAGATCTAGTTCTAGCGAACTGGAACAAC	Core	Orange
13[133]	15[146]	CAGTAACAGAAAAAAACGCGAGAAAACTCAGAAAAGAAGCAA	Core	Orange
19[182]	15[181]	ATTATACGTGATAAAACTAAAGTAGCTCAACATGTAAGCCCG	Core	Orange
0[139]	30[133]	TGGGCGCAGCTTGAGAGGTTG	Core	Orange

17[161]	14[161]	TTAGTTACCTCAAAAAAAGATTAAGAGGTTTTCGCCTGATTG	Core	Orange
6[55]	27[48]	GACCTGATTGAATGGAGGTTTTGAAGCCGCTACAAAAGAAACCTTTTTA	Core	Orange
26[237]	31[244]	CACTGAGTCATTTTTTATTCTGTGCCCGTCCCTCATTCATAATCAAAAT	Core	Orange
25[98]	8[98]	TTTGCCATATCAGCGCCATTATATTAAC	Core	Orange
0[76]	29[83]	GAACGTGTTTTTTGAAGGTAACAGCGCCAAAGACA	Core	Orange
31[161]	2[161]	TAGCGTCCCACCAGAATTCGTCTTGTTA	Core	Orange
25[224]	8[224]	AGTTTTGAACTTTCTGCATCTTTTGTTA	Core	Orange
5[224]	23[223]	GGCTGCGCCGCTTCTGGTGCCGTAACCGAACAGTTTCAGCAG	Core	Orange
15[210]	9[216]	GAGCTTCTTAGAGCTTCCATAATTTGGGCAAATCACTGAGAGATCAGAA	Core	Orange
24[153]	28[147]	AAAATCTAGAAACGAGACGGGGGTGTATGGATAAG	Core	Orange
6[132]	3[132]	ATTTACAGATTAGTGGATGTTATTGTCA	Core	Orange
29[119]	5[118]	ATGGAAAGGCATGAGTGCTGACTTCTAAGTGGTTGAGCAATA	Core	Orange
6[237]	2[231]	TCCGGCACAACTGTGGGGATGGTAAGCAATAAATC	Core	Orange
8[55]	11[62]	AAAATCTACCCTCAGAATCATAGCTAATGCAGAACGTAATTCATGGCAA	Core	Orange
29[168]	0[161]	GCTTTTGAGAACCAAGACTGTAGTTGCAGCAAGCGGCAACAGAACGCGC	Core	Orange
20[202]	14[203]	GACCAGGGACGTTGGGAAGAAATTACGAAGTTTCATTAATTG	Core	Orange
6[195]	0[189]	TGCAACACCAGAACTTTCCCACTGGAGTGAGTAAACTGTGTGTCGTGCC	Core	Orange
14[97]	16[84]	AGAAGATGTAAATCTGAAAACATAGCGA	Core	Orange
8[111]	4[105]	CGGTCAGAAAATACAGTCACACCGTTGTTGAATTC	Core	Orange
25[161]	8[161]	GTTTAACATTTTTTACAAACGTTCCTGT	Core	Orange
31[196]	2[203]	TCGGTCATAGCCCCCCACCCTTATCCGCCTGGTTG	Core	Orange
9[147]	4[147]	TAGAGCCTTTCATCGATTCTCCTCAATCCCTGAGTTTTCTCA	Core	Orange
28[69]	31[69]	ATAAAGGGGTTTACATATTGAACCAGTA	Core	Orange
9[161]	5[174]	GATAATACATTTAATCTGGCCGCGGATTGGAAATATATCGGC	Core	Orange
25[56]	8[56]	CTGAATCCTTGCGGGCTATTACAAATGA	Core	Orange
20[76]	14[77]	CGACAAACCAGTAATAAGAGAAACGCTCTAAAACACAAACAT	Core	Orange
29[224]	4[224]	AGTAACAGAAACATGCCATCTTGCTGCA	Core	Orange
19[119]	14[119]	GAGATGGTAGTATCGTCAGATAATTATT	Core	Orange
11[63]	6[63]	TTCATTAGCGGAACTCTGGTCCCAGCAGGTCTTTAGATAGAA	Core	Orange
8[216]	24[196]	TCATTTTGCGCATCGGAAACCAGGCAGCCAGCCATGTTGGTGATAGAAA	Core	Orange
11[168]	19[160]	TGTAGGTGTGAGGATTTAGAAGTATTAGATGCCTGTGACAAGTACCTTA	Core	Orange
6[181]	9[188]	CGCTCATGACCGTAAATTGCGGCTTGCAGGGAGTTTAAAACGATGTCAA	Core	Orange
29[56]	4[56]	TTCATATTGGCAACCGCGTACTTAAAGG	Core	Orange
42[125]	29[118]	GTTTGATATCAAAAAGATAGGGTTGAGTTAAAGGGATTGGCCAGCCAGA	Core	Orange
5[63]	0[63]	TGAGGCCAGGCCGATATGGTTTGTAGCGAATCAAGGACTCCA	Core	Orange
8[97]	20[98]	ACCGCCTAAGGAATCACTCATCTGAACAAGAAAAAGAATAAG	Core	Orange
26[118]	8[119]	CAGAGGGAATAAACAGCCATAGAGCCTTACCACCAAAACAGA	Core	Orange
27[182]	6[182]	ACCCTCAACAGAGAGTAATATGGAAAAA	Core	Orange
16[181]	21[174]	ATTCATTGAATCCCATTTCATGTTAAATTTAAGAAATCAAGAACCAAGC	Core	Orange
20[125]	17[118]	TAACAAAAAAAATTGCAAAATTAAGCAAGTTTAACATATGCGAAATCCA	Core	Orange
0[160]	28[161]	GGGGAGAGAGCTCGAGCCGCCAAGCGTCATACATGGCTCAGT	Core	Orange
11[98]	17[97]	CCTTTATTCAGAGCATAAAGCAGAAATTACCAGTAACTATAT	Core	Orange
18[195]	23[202]	CGACCGTCAGTCAGCGCATAGAACGGAGACCAACCAAAGGCCGCTTTTG	Core	Orange
11[182]	17[181]	CAAAAGGTCAATTCTACTAATAATATGCATAAGGCCTTCTGA	Core	Orange
25[140]	8[140]	CCAAATACCAAAAACCCGTCGAACATTA	Core	Orange
19[56]	16[49]	GAGGCATATTGAGACATATCATAATTACATTTAACCAGTACACAATAGT	Core	Orange

17[56]	15[62]	ACCTTTTGAAGAGTTAAATCA	Core	Orange
3[147]	28[133]	TCCCGCCAAAGACGGAGGAGGGAAAGCCACCTTATGAGAGGG	Core	Orange
1[210]	29[202]	TCACAATGCTTTCCAGTCGGGAAACCTGAAATTGTCAGAGCCAATAAGT	Core	Orange
20[216]	24[210]	ATGAACGCTGATAAACGTAATGTCACCCTCAGCGG	Core	Orange
2[132]	39[132]	GGCGAACGATTTAGCAGCCCGGAATAGG	Core	Orange
3[105]	0[91]	CTTAAGTAGGGAAGGGAACCCGTTGTTCCAGTTTGGAACAAG	Core	Orange
27[98]	6[98]	AACGCAAGCGCTAAAAATTAACGACCAG	Core	Orange
27[140]	6[140]	CGGAATAAGAATTATCACTTGGTCTGAA	Core	Orange
17[182]	13[195]	CCTAAATCATAAATAAAGACTTCAAATATAATGCTGTACGGT	Core	Orange
16[244]	20[231]	AGAGGGGGTAATAGGTTTACCCTAATGCACTAACGACCAACT	Core	Orange
20[90]	22[98]	CTGACATATAAGTCCGAGAACCGTGAATTTCTTAAACAGCTTAGTACCG	Core	Orange
28[132]	42[126]	TTGATCTGCGCAGTAGGCAGGACCGTAAAAATCCT	Core	Orange
1[147]	31[160]	GGGTACCGGCGGTTCACCAGTGAGACGGGTCCACGTTGCCTT	Core	Orange
1[231]	29[223]	GAGCCGGATTGCGTTGCGCTCACTGCCCTCCACACCACCCTCTGCCTTG	Core	Orange
12[90]	7[90]	TAAATCGTTTTGCGTTTGAGTCAGTTGAGCAACAGAGCCCTA	Core	Orange
18[153]	20[161]	GAATCATTGTGAATAACCGGATATTTGACCCCCAGCGATTATGTAATCT	Core	Orange
17[196]	15[209]	TTTGACATAGTAAGCGTCCAATTAATTC	Core	Orange
25[105]	31[111]	GTTACAATAATTGATAATAACTCCTTATTCATTAATTGATATAATGAAA	Core	Orange
6[90]	3[90]	AGGGACATCTGTCCTCAGAGCGTATAAC	Core	Orange
23[49]	10[56]	CAGATATAGAAGGCATCGTAGATCAATAAAAGAAA	Core	Orange
20[132]	24[126]	ATCAACGGAGCATGTATCATTAATGACACCAAAAG	Core	Orange
12[195]	7[195]	GGTGGCAGTGAGAAGAGAATCTCATATGCGCCATCAGGTCAC	Core	Orange
21[140]	12[133]	CAATCAATATTCATATATTTTTACAGGCAAGGCAA	Core	Orange
27[119]	6[119]	CCAAAAGACAAAGTCTTCTTTTTGGCAG	Core	Orange
28[83]	26[70]	TAGAAAACGAGCACGGGAGCTAAACAGGACCGAGTGAATTGA	Core	Orange
22[174]	28[168]	CAAAAGACGCTGAGAATAATAGTCAAAAATAAAAAGGTTTAGGGGTTTT	Core	Orange
18[69]	23[69]	GGGCTTATTTCGAGAGGTAAAGCGCCTGTATTTTCTTATCCG	Core	Orange
17[77]	13[90]	GGTTGGGTAGCTTAATAACCTTGCTTCTGATGAAAGAAATAA	Core	Orange
39[133]	1[146]	GTGGTTTCAGGCGATCAGTAGCGACAGATTGACAGCGTCCCC	Core	Orange
19[224]	14[224]	AAAACGAAGATACATGATTCCTTTTTGC	Core	Orange
20[83]	17[76]	AAAGTACGTAATACGTTGTACCAAAAACTTGCACGAACAGTACGGCTTA	Core	Orange
15[63]	12[56]	ATATATGACAAAATAAATTATATCAATATAATCCT	Core	Orange
24[195]	8[203]	GGAACAACTCAGACAGCCCTCATAGTTAAGTGAGATAGATGGTTAACCA	Core	Orange
29[133]	4[140]	CTCTGAATTTACCGTGCCGTCGACAATGGGAGAAG	Core	Orange
19[245]	15[244]	ATTATTAAATACCAAGTAGATGCTCCTTTTGATAAAAACTCC	Core	Orange
13[126]	7[132]	GAATATAAGAATTATTTAGAACTCGTATGAGCACTGCAGATAGCAGAGA	Core	Orange
20[209]	18[196]	GTGTACAAGACAGTGCGCGAGCTGAAAAGTCTGGAGGAATAC	Core	Orange
5[77]	23[76]	AAAAGAGTTCTGGCCAACAGAATGCGCGAGCGAACGTATTCT	Core	Orange
11[231]	19[223]	TTCAACCCAAAGGCTATCAGGTCATTGCCCATCAAAGGACAGCGTTAAT	Core	Orange
6[118]	23[125]	ATTCACCCGAACGATAATTGTATAGTTGCGCCGAC	Core	Orange
2[223]	26[224]	CGAACTCCGGCCCTGAAAGTACAGAGCCACCACCCTTTCGTC	Core	Orange
21[238]	12[245]	TGCTCCATGTTACTAGGGAACCTGATAACGCAAAT	Core	Orange
4[174]	39[174]	AAACGACTCTAATCCGTGGTGAATCATGATCGGCCCTGATTG	Core	Orange
17[98]	14[105]	GTAAATGGAATCCTGTCGCTATTAATTAATTACCT	Core	Orange
1[91]	30[98]	TAAAGCACATCAACCGATTGAAGCAAGGCCGGAAACGTCACCTCACAAA	Core	Orange
19[161]	12[168]	TGCGATTAAGAATAAACAATAACGGATAAGTAGTA	Core	Orange

3[161]	25[160]	CCCCGCTGGCCAGTGCCAAGCAGAAGAAGCGCATTATTTTTT	Core	Orange
26[195]	31[195]	TTCCATTGAACCGCAGAGAAGTACTGGTACCACCCGGCATTT	Core	Orange
6[160]	22[161]	TTTGACGCGTGGGACACGTTGCCGATATATTCGGTATACACT	Core	Orange
11[161]	17[160]	AGTAATGGCATTAACATCCAACGGGAGAAACACCGATATATT	Core	Orange
2[160]	27[160]	CCTCGATAAAATAAACCAGGCCACCGTA	Core	Orange
2[118]	27[118]	GAAAGGAGTCCTTATTAAGACGGAATAC	Core	Orange
14[118]	14[126]	CATTTCAATGGTCTTTACCCTGACTATTAGAGGCG	Core	Orange
2[202]	27[209]	GTGTAATGACTCTACTCCTCACACCCTCAGAACCG	Core	Orange
2[76]	26[77]	TGGCAAGGCTTTGATACATACAACAAAGTTACCAGCCCACAA	Core	Orange
14[76]	17[69]	CAAGAAATGAGTGAGATTAAGACGCTGATAACCTC	Core	Orange
14[202]	17[195]	CTGAATATCGCGTTTACTGCGGAATCGTTTAATGG	Core	Orange
8[160]	23[153]	AGCCAGCGTCAATAAAAACACTCATCATCGCATAA	Core	Orange
8[76]	21[83]	CTGAGAGAGTTGGCCCGTTTTTTTATCAACAATAG	Core	Orange
8[202]	21[209]	ATAGGAATACCCCGCGAAGGCATTTGTATCATCGC	Core	Orange
14[160]	17[153]	CTTTGAATGCATCATGCTTTAAACAGTTTTTTCAA	Core	Orange
0[62]	44[35]	ACGTCAAAGGGCGATGAACCATATTCATTAGAAAACTCTGGTTAACGTGTCTGGGC	Bottom OH	Red
12[244]	38[224]	GGTCAATGCGAACGCATTCAAAGACGACCTCTGGTTAACGTGTCTGGGC	Bottom OH	Red
0[188]	44[161]	AGCTGCATTAATGAGTCATAGCGCCACCATGATACCTCTGGTTAACGTGTCTGGGC	Bottom OH	Red
17[84]	48[77]	TTATATATAAAGCCATGAGAAACACCAGCTCTGGTTAACGTGTCTGGGC	Bottom OH	Red
4[139]	40[119]	CCAGGGTAATAACAACTGAACTCCCAATCTCTGGTTAACGTGTCTGGGC	Bottom OH	Red
0[251]	44[224]	GCTAACTCACATTAAAGCATAAACCGCCTATAAACCTCTGGTTAACGTGTCTGGGC	Bottom OH	Red
10[188]	48[161]	GATGAACGGTAATCAAAGATTGACCTTCCTGGCTCCTCTGGTTAACGTGTCTGGGC	Bottom OH	Red
10[55]	48[35]	CCACCAGTCAGATGTGTCCAGTTAGGCACTCTGGTTAACGTGTCTGGGC	Bottom OH	Red
4[104]	40[77]	ATGCGTCGTTAGAAATCACGCTATCAGAAGCCTAACTCTGGTTAACGTGTCTGGGC	Bottom OH	Red
8[223]	34[203]	AATCAGCAAGCCCCGTAAAATATTGTGTCTCTGGTTAACGTGTCTGGGC	Bottom OH	Red
2[55]	32[35]	TGCGCGTTACAGGGATATAAAAGAAAAGCTCTGGTTAACGTGTCTGGGC	Bottom OH	Red
2[181]	32[161]	TAAGCTATATTTACATTAGCGTACCGCCCTCTGGTTAACGTGTCTGGGC	Bottom OH	Red
4[244]	40[224]	GCGAAAGTGGGAAGACCGTAATCCAGACCTCTGGTTAACGTGTCTGGGC	Bottom OH	Red
10[146]	48[119]	AAACAATTCGACAACCCTCATTACCCAATCAACTTCTCTGGTTAACGTGTCTGGGC	Bottom OH	Red
28[48]	36[35]	AGAAACGCAATCAATAAAGGTGGAATTAGAGCCAGCTCTGGTTAACGTGTCTGGGC	Bottom OH	Red
12[223]	38[203]	TATTTTCTAACAGTTAACGCCCTATCATCTCTGGTTAACGTGTCTGGGC	Bottom OH	Red
25[126]	32[119]	TTATTTAACCCTGAAAATAAGTATAGCCCTCTGGTTAACGTGTCTGGGC	Bottom OH	Red
12[55]	38[35]	GATTGTTAACCTACATCGCCAAGAGACTCTCTGGTTAACGTGTCTGGGC	Bottom OH	Red
29[210]	36[203]	GGGTCAGAGAACCGCTTATTAGCGTTTGCTCTGGTTAACGTGTCTGGGC	Bottom OH	Red
8[139]	34[119]	AATGTGAAACAACTCTTTCCTTAGAAACCTCTGGTTAACGTGTCTGGGC	Bottom OH	Red
4[55]	40[35]	GATTTTAGTTTTTATAAGAGCTTTTATCCTCTGGTTAACGTGTCTGGGC	Bottom OH	Red
4[223]	40[203]	AGGCGATCCATTCAACCAGTAGATCTAACTCTGGTTAACGTGTCTGGGC	Bottom OH	Red
35[98]	21[125]	GTTTGGTTGTGGTGTATAAATTGGTGAGGTAAAATACGGGTATCCATCCTAATTTAC	Top OH	Green

Supplementary Table 2. List of ssDNA staple sequences for folding the top nano-brick. The start and end columns denote the helix[base] positions at the 5' and 3' ends of the DNA staple strands in the cadnano design (Supplementary Fig. 4). Color is also with respect cadnano.

Start	End	Sequence	Type	Color
42[83]	14[87]	AAATCACTGTGTAGTGCGAATAACAGTTTCAGCGGCCAC	Core	Green
13[49]	45[55]	AACACTGAAGTTTTCTGTATGATCTCCAATCGGTTATATGTAAGGAAGA	Core	Green
13[74]	45[76]	AACCATAGTTAACTTTCAATAATTCGAGGTGCGTAAAAATTTAAA	Core	Green
19[126]	40[126]	AAGACACAGCGCCAGGAAATTGAGCCAGTGATGCATTAATTT	Core	Green
21[70]	47[66]	AAGAGGCTTGAGGAGGCCTCAATTGACCGTAATGGATCA	Core	Green
31[119]	62[119]	AAGCAAAACCTCCCGAAATGGTTCTGGC	Core	Green
54[104]	54[105]	AAGTGTATGGGCGCCACAACTAATAGATAAATCCTTTGCATA	Core	Green
46[76]	50[80]	AATCAGCTTAAATGTGAGCGAACGGCGGGGAAGATGCGC	Core	Green
23[70]	49[66]	AATCATAACGGAGAGCAAGGCTCGCCATTCAGGCTCAGT	Core	Green
39[98]	15[104]	AATCGGTGGCATTTTCGGTTTAGTGAGAATAGACC	Core	Green
65[63]	10[70]	AATTAACCGTTGTAATCAGGTAATGCTTGGATTAG	Core	Green
47[67]	41[59]	ACATCATTTTAGCAAATCTAGCATAAGGCTAATGATATTCAACCGTTAA	Core	Green
23[91]	49[90]	ACCAACTCCAAGCGACGCCAGGGAAACCAGGCAAACGCACTC	Core	Green
15[126]	39[115]	ACCAATGTAGCGTCAAATAAGAGTATCATATGCGTTATACAAACCG	Core	Green
32[90]	64[98]	ACCATAAAGCGAACCAAGCATGTAGAAATCATCGTATAACATGGAAATA	Core	Green
57[119]	30[126]	ACCCTCAATCAATAAAATCTACGATTTTTTCCAGACAGCTACTTAGCGA	Core	Green
21[112]	49[104]	ACCGAGGGTAGAAATTATTTGTTTACATCGGGAGAAACAATTCTTTCCG	Core	Green
12[111]	37[118]	ACCGCCAATTAGCGAAAGTACGTAGGGCTTAATTG	Core	Green
39[116]	43[125]	ACCTGACCTACTATATGCTCCGGCAACATAGCGATAGC	Core	Green
42[132]	15[125]	ACTACCTTTTTAACTAAATGCCAAAATCAAACGTC	Core	Green
64[44]	10[49]	AGAAAAGAGTCTGTCCATTATAGTATATTCATGCTCAG	Core	Green
14[86]	35[80]	AGAAGAGCATGGGCGCGAGCTGAATAGCTATGTACGGTAACA	Core	Green
37[119]	32[119]	AGAATCGTAATAAGTAAACAAAACAAGAGCTGTCTACAAGCA	Core	Green
30[90]	11[90]	AGAGGCTTTCAGAAAACGAGAAACTGAGACTCCTCTACCGCC	Core	Green
12[58]	63[62]	AGAGGTGTATGGGGTTTTTGAATCGAGGGGGATAACGT	Core	Green
61[102]	28[91]	AGCACGAACCACATCAAGTTTTTTCGGGCGCTAGGGCCGCAGGTA	Core	Green
47[56]	51[62]	AGCTTTCGATAGGTACGACGAGCGCAACAAGGGGG	Core	Green
23[56]	57[69]	AGGCGCAAAGAGTACATTCAGCCCTTCACAGGCGAAAATCCT	Core	Green
59[42]	64[45]	AGGGCGACTAAAGGCGGCGAAGCGCTTATTTGACGCCTG	Core	Green
60[76]	55[76]	AGGTGCCGGCCCACAGTGTTGGTTTGATGCAACAGAACGCGC	Core	Green
6[117]	7[117]	AGTGTACTGGTAATAAGTTTTTAATGCCCCATACATGGCT	Core	Green
55[137]	23[132]	ATAAACAATTCGACAACTTTTAAAAAGAATTTGAAATA	Core	Green
29[56]	60[56]	ATCATAATACCACAAGAAAGGACTAAAT	Core	Green
49[67]	41[66]	ATCCTAAAGATCGTCACCGCCCACTTGCTTTTTTTCACCAAT	Core	Green
65[133]	34[129]	ATCGGCCCGCACTCTAGAAGGAGACGATTGGCCTTGAGCCGCAGTC	Core	Green
41[60]	38[63]	ATGGTTGAAAGGATTTTGCTAAACAGCGTAAAAAATTACATCAAT	Core	Green
15[105]	39[97]	ATTAGCATTTCATCTTGAAATATTCTTACCAGTATAAAGCTGAAAGCTA	Core	Green
43[77]	19[76]	ATTGCCTCGGTAATAATTTCTATGACAACAACCATCCTCAGC	Core	Green
45[119]	21[125]	ATTTGAAGAAACAAAAACGCAAGCAAACAAACGCA	Core	Green
22[125]	56[119]	CAAAGTTGAAACAAGAGTTAACTGAACAATATCTT	Core	Green
40[146]	14[140]	CAAATATATAAGAAATCAAGT	Core	Green
62[118]	26[105]	CAACAGATACGTGGTTGCCAGATCCAAAAAGCGCATTAGAAT	Core	Green

46[114]	51[122]	CAACTGATTGCTTTGAAAGTACCTCACGTAAGAACCTAAGAT	Core	Green
11[109]	14[112]	CACTATCCCACCAGACGACGACAAAGAATATTTTGCCAAGCGCGT	Core	Green
48[132]	43[132]	CAGTAACTACCAAGAGATGATTTACCTTCTGTAAATTAGATT	Core	Green
59[126]	65[132]	CAGTATTCCTAAAAAATATTTAGGGACAATTATTTCAGCCATTCAAACT	Core	Green
42[146]	15[146]	CATAGGTTCGCAAGCCATTTGCGATAGC	Core	Green
40[125]	10[119]	CATCTTCGTGTGATAGACTGTTCTTTTCCCGCCACACCACCAGATATTC	Core	Green
62[62]	25[52]	CCACCACACCCGCCCGTGGCGTTCAACTAGAAAAATGCGATTAGCT	Core	Green
8[117]	9[118]	CCAGTAAGCGTCCGGAAAGCGCAGTCT	Core	Green
64[97]	32[91]	CCCAGGAGGCCGATTTCTTTGATTAGTAAGGAATG	Core	Green
13[98]	37[90]	CCCCCTTCCCTCAGAGCCACCTCAGAACCCTGTAGAACTAAAATTTTCA	Core	Green
12[48]	38[52]	CCCTCATGTACCGTTGATTCCATTAGATACATTTCATAG	Core	Green
57[84]	25[97]	CCGAAATCCGAGATCAACATTATTACCAGAGATGGCCAGAAC	Core	Green
12[69]	38[73]	CCGCCACTCACCAGAGTTTCAGTCAATAACCTGTTAAGG	Core	Green
34[90]	11[97]	CCGGAAGTTTAAATATAAGTAATTCTGTTCCTAATTTACGGAACCCACC	Core	Green
60[107]	65[111]	CGAGTAAGAAGATAGAAACGCTCACGCTCATCACTTGC	Core	Green
11[133]	37[132]	CGCCAGCCGCCTCCCTCAGAGATAATCAGAGCCAGCCATATT	Core	Green
60[55]	54[49]	CGGAACCAAAACCGAAGAGTCGCCCCAGCCGCCTGAGCTGCACGTTGCG	Core	Green
23[49]	20[42]	CGGAACGTGATAAACTGGCGATGTTGGGAAGGGCGGCCAGTTAGTTTCC	Core	Green
20[90]	40[84]	CGGCTACGACAGCAGCCGACATAAACAGAAGGAATGTAAAGAACATTAT	Core	Green
54[48]	23[48]	CTCACTGATGGTCACAAGAACACTTAGC	Core	Green
32[62]	29[55]	CTGACTATCACGCACGGTACGCCAGAATAGCACGTTAATAGTCAACACT	Core	Green
65[112]	11[108]	CTGAGTAAGCCGTTCAATAGCACAAACAAATAAATCCGC	Core	Green
34[128]	16[136]	CTGCATGTTCAGCTAATCATTTTCAAATCACTTGCCTTAAACCATGGAA	Core	Green
62[72]	24[70]	CTGGAAAGCGTTGAGATACGAACTTAATCATGGCTTGCGGCTGAC	Core	Green
57[42]	30[42]	CTGGTTTCACTATTGTTGGGAAATGCAGGTAAGAGAAAATGT	Core	Green
32[69]	12[70]	CTTTACCGCGTTTTAATTCGACAACAGGCTCAGGACTCAGAA	Core	Green
28[90]	61[101]	GAAAGATTAAAAACCAAAATTAAATCAAATAAACATGAA	Core	Green
40[83]	11[80]	GACCCTGAAGCCTCCAGCCCTTACAACGCGCCACCGGTT	Core	Green
62[97]	30[91]	GACGCTGGCAAGTGGCGGGAGCTAAATACCTAGCG	Core	Green
42[135]	18[140]	GAGAAGACGCTGAGAAGGAATAACAAAGGTGTCATATG	Core	Green
47[98]	19[104]	GATAACGGATTCGCGAAAACAAAATTAAGAAGGTG	Core	Green
10[69]	13[73]	GATTAGCCACCGTATCAGGATTAATGCTGTAGCTCGTCTGGATACA	Core	Green
19[105]	47[97]	GCAACATACATTCAACCGAGATAGTTGCTCGGAACATTCGCACCCGTCG	Core	Green
23[133]	20[126]	GCAATAGAGCAGATCATCAATTAATGGAAGGGTTAAACAGAAGTATGTT	Core	Green
49[105]	21[111]	GCACCAAATACATACATAAGGCATCTTTGACCCAA	Core	Green
7[84]	8[98]	GCCCGTACCTATTAAGTATTAAGAGGAGCCAGAATTGCCTAT	Core	Green
42[96]	42[97]	GCCGGATGGAGCAAAAGAATCCTTGAATTAGGTTGGAGAAAG	Core	Green
41[67]	12[59]	GCCTTTGCGGGAGAAGCAATTAGCCGATCTAAGTTTCGCCTC	Core	Green
25[53]	22[45]	GCTATCTTGATAGCTGTCTAGAGGATCCCCGACGCCAGTTGT	Core	Green
48[90]	42[84]	GGAACAAGTAACAATTAAATTCGTTAATCGATGAAGAGAGTCGACAGTC	Core	Green
17[105]	40[91]	GGAGGGAGCACCATTATATAAAATTTAATGGTTGTACCAAAA	Core	Green
59[63]	64[63]	GGGCGATGTAAAGCAAGGGAACGCGTAAGCTTTCCACAGGAA	Core	Green
25[137]	22[126]	GTCAACCCACAGTTTGAACCAGAAGGAGCGGGGCAATTAGCCGAA	Core	Green
44[62]	47[55]	GTCAATCTATCAGCGCATAACCGATATACCGCTTTATAGGAATGTAGCC	Core	Green
31[42]	12[49]	GTCATAACAGAAGCGCCCGAAAGACTTCTACCTTTCGGAATAGCCACCA	Core	Green
52[62]	25[69]	GTCGACTTTCCTGTTTAATTGTTAATGAATCGGCCCTGATTGTGAATAA	Core	Green

18[139]	47[146]	GTTTACCCACGGAACAAAAGATTACAAAATCGCGC	Core	Green
45[98]	17[104]	TAAATTACATTTAATAATTTTCCCTTCACCTTGAG	Core	Green
37[133]	42[136]	TAACAACCCTGTTTGCGTTAAATTTTAGAATCCAACTGA	Core	Green
62[139]	25[136]	TAATAAATTGAATGAGCGTCTTTGTTTAGAGAATACAAA	Core	Green
24[97]	51[90]	TACAGACCACAACAAAACGACGGCCAGTTTGGGTA	Core	Green
10[48]	36[49]	TACCAGGTATAGCCAATTGCTTTAGAGCTTAATTGTAACAGT	Core	Green
63[74]	60[77]	TAGAAGAAGTTTACCAGACGACGATCATCAGAAAGGAGGGGGTCG	Core	Green
11[81]	62[73]	TAGAAGAGAATAAACAGTTTGCAAAATCAGATAGCGGTCACG	Core	Green
38[51]	43[62]	TAGGGCAAAGCTTTATTATATATTTTCTAGCTTGAGAGATCTACA	Core	Green
31[126]	34[133]	TCAGATAATCGAGATTCCTTATCATTCCATAGATA	Core	Green
44[125]	46[115]	TCGTCGCTATTGACAAGACAAAAGGGCGATAAAAGACAT	Core	Green
55[42]	26[42]	TCGTGCCGCCCTGAGTAACAATTAAGAA	Core	Green
38[62]	33[62]	TCTACTAGCAAATGTTCCATACTGAATATAGAGAGAAATATC	Core	Green
58[111]	31[111]	TGAACAATATCCCATTACAAAGATTAGTGGGAGGTCCGCGCC	Core	Green
54[76]	23[69]	TGAGCTAACTCACAGTGAAATCTTCATCGACGGTC	Core	Green
60[139]	55[136]	TGATAGCAACACCGAAATGAATCTGGTCGAAGGTTGATA	Core	Green
38[72]	43[76]	TGGAGCAATATAATACTTGAGTAACATCAATTCAGGTC	Core	Green
26[104]	60[108]	TGGGCTTTATTATTATCAAAAGCAGAAGATAAAACATAC	Core	Green
35[81]	63[73]	TGTCAAACTCGCTTCAAATCAAAAGCAATACTAAAGGGATTTTAGTCGT	Core	Green
53[70]	21[69]	TGTTATCTTGCATGCCTGCAGATGTGCTTTTGTATAAAACGA	Core	Green
16[135]	19[146]	TTAATTCATTCTTGCTTTTTTAATGGAAACAACCTGAGTAAGTTT	Core	Green
64[139]	29[139]	TTACCGCACATTGGAGGCGTTAATTTTA	Core	Green
41[91]	45[97]	TTCAAAAGGGTGGTTAAAGGAACAACTACTTGATAAGAGAATATTTTGT	Core	Green
37[91]	13[97]	TTCAACGCTCAACACGACAAAAGGTAGCCACATAG	Core	Green
58[69]	31[69]	TTCCAGTTAATAAATTAGGAACCCTCGTTTTGCCACCCCTCA	Core	Green
8[97]	6[84]	TTCGGAATAAACAGTAACGGGGTCAGTG	Core	Green
56[97]	23[90]	TTCTTTTTGCGTATAAGCCTGGGGTGCCAATTCCACAGGCGCCGAACTG	Core	Green
45[77]	22[77]	TTGTAAATTTGTTAAGCGAAAAGAGGCTAAAAGAAAAGTACA	Core	Green
45[56]	22[56]	TTGTATATTAACCATGCGGGACTTTTTCCCAACCTCATCGCC	Core	Green
32[111]	12[112]	TTTATTTCCAATCAATAATCGAAAATAACAGAACCCCTCAGA	Core	Green
48[41]	51[41]	TGGGCGCTGCATCTATCGGTGCGCTATT	Core	Green
56[111]	52[105]	ACTAAGGAACGGGAGAATTAAGCCCAATCCGAACGATCATATTCCACGA	Core	Green
23[105]	50[105]	AGGACTAAGAGCAAACCAGAAGATTATCCCATATCAAGCTTC	Core	Green
23[77]	57[83]	AGGGAACATAGGCTCCTGACGGAGACGGGGTGGTT	Core	Green
58[83]	57[97]	AGGGTTGTACGTGAACCATCACCCAACAGAATAGCCGGCAAA	Core	Green
56[132]	59[125]	ATCTAAACCCTGAAACATAAAAACAGGGTAAGAAAAAGCATCAGGCGGT	Core	Green
52[104]	25[104]	CGTTGTATACGAGCCGGAAGCAAAGATGAACGGTGGAGTAGT	Core	Green
65[119]	29[118]	GAAGAACTGCAACAGGAAAAAATCGTCTGACTTGCTGCTATT	Core	Green
51[123]	55[132]	GATAATTATCTTATTAATCGTATTTAGAGCCGTCAATA	Core	Green
55[84]	27[83]	GGCGGTTCACCAGTAGAAACATTTAATTTCAACTTAACGGAA	Core	Green
22[44]	48[42]	GTCCCACTACGAAGGCAATGAGGATGAGGGGCACGTTGGTGTAGA	Core	Green
56[104]	56[112]	GTGGTTTATCCCTTATCTCAAATATCAATAGGAGC	Core	Green
50[104]	23[104]	TGGTGCCGGTTTTCCCAGTCTGGCCAGCGATTATATTGAAAG	Core	Green
26[69]	59[62]	TGTGAATTACCTTATCTACGTTTGGAACTCTATCA	Core	Green
29[119]	58[112]	TTGCACCGCCTAATCACAGACCATCGCCATTAAAAAGAGGTGACCTTGC	Core	Green
15[112]	45[118]	AGGCCGGACCAGTAAGGTAAATATTAATCAATTTC	Fluorophore Core OH	Yellow

21[126]	48[133]	ATAATAACGGAATATTACGCAATAAAGAGAATATA	Fluorophore Core OH	Yellow
50[79]	55[83]	CATGATTAAGGCCAAGCCGCTCACTAATGAGGGGGAGA	Fluorophore Core OH	Yellow
22[90]	48[91]	CGAAACATACACTAAAACACTGTAGCAACAGCCAGCTCCGTG	Fluorophore Core OH	Yellow
11[98]	62[98]	CTCAGAGCCTCATTATCATTATTTGAAGCATTTTGCCCTTCT	Fluorophore Core OH	Yellow
33[140]	64[140]	AAGAACGCAAGTACTTGCTGGAACAATA	Fluorophore Neighbor OH	Orange
47[147]	42[147]	AGAGGCGTTCAATTGTACATATGTGAGTAGTCAATTCAAAAT	Fluorophore Neighbor OH	Orange
26[41]	59[41]	CTGGCTCTCAGGACAAAGAACACGTCAA	Fluorophore Neighbor OH	Orange
52[41]	55[41]	GGTACCGCGTAATCCCCGCTTAAACCTG	Fluorophore Neighbor OH	Orange
29[140]	60[140]	TCCTGAAGCTAACGGCTATTACGCGAAC	Fluorophore Neighbor OH	Orange
15[147]	38[140]	AGCACCGCGACAGATAAACACATTACTAGAAAAAG	Neighbor	Black
20[41]	23[41]	ATTAAACCGTAATGGAAATCCCCATGTT	Neighbor	Black
19[147]	40[147]	ATTTTGTAGAAAATAATTATCACTTGAGACAAAGAACTTTTT	Neighbor	Black
13[140]	10[140]	CGGAACCCCGGAACATTGACAGCAGGTC	Neighbor	Black
24[41]	57[41]	CGGATATAATCAACGAGAGTTGTCCACG	Neighbor	Black
31[140]	62[140]	CTTATCCGAACGCGCAGATTCCGACCAG	Neighbor	Black
37[140]	34[140]	GCCAACAGCAGAGGGCAGAACATCAACA	Neighbor	Black
65[147]	64[147]	[DIG]<br>TTTTTTTTTTTAATAGTGAGGCCACCGAGTAAGTTTTTTATAATCATCCAG	Digoxignenin OH	Brown
11[21]	10[21]	[DIG]TTTTTTTTGGAGGAGAGGGTTGATATAAGCGGATAAGTGCCGTCGTT<br>GAG	Digoxignenin OH	Brown
20[34]	21[34]	[BtnTg]<br>TTTTTTTTTTGGGTAGATTAAGACTCCTTACCCAAAAGAACTGGCAAAATA	End Biotin OH	Purple
		TTTATACACCACAACCAAACCCATCTCAAATATCAATAGGAGCACTAAGGA<br>ACGGGAGAATTAAGCCCAATCCGAACGATCATAT	Attachment OH to Platform	Not Shown on CaDNAno

Supplementary Table 3. List of ssDNA staple sequences for folding the nano-hinge. The start and end columns denote the helix[base] positions at the 5' and 3' ends of the DNA staple strands in the cadnano design (Supplementary Fig. 6). Color is also with respect cadnano.

Start	End	Sequence	Type	Color
31[119]	15[132]	AAGTCCTATTTTAATACGAGCCGGAAGCGAGCTAATTAAACC	Core	Green
34[181]	30[175]	AATCATACAGGCGCTGACAAGTAAGGCTAATTGGG	Core	Green
22[174]	23[160]	ACCACCCTTTCGTCGCGTAACGATCTAAAGTTTAAACCGTAA	Core	Green
19[161]	19[160]	ACTCATCTATCATCGCCTGATAAATTCCCCATACACTAAAAC	Core	Green
25[119]	28[119]	AGAAGAAGGAAATACGGAACCAACCTTGGGATTATCTTTCCA	Core	Green
7[168]	4[161]	AGACGTTGCGGAGTGAGAATAATTTTTTCACGTTGCAGCTTGCTTTCGA	Core	Green
18[160]	0[154]	AGAGGCAAAAGACTTAAAGACTTTTTCATACAGAG	Core	Green
4[139]	3[146]	AGCGGTCCGTACTATGGTTGGAATTGCGGCGCTGGCAAGTGTAAAGGAG	Core	Green
7[126]	5[125]	AGGAGGCACGTATAACGTGCTGCCGCTA	Core	Green
26[132]	8[126]	AGTGAATAGAGCCAACAGGAGCTGAATTCAAACAA	Core	Green
13[119]	34[126]	ATACAGTCCAATCAACGGGTACTCACATTCGGGAAACCTGTCCGGGGAG	Core	Green
34[139]	30[133]	CAACGCGGTGCCAGAATGAGTATAAAGTAATTACC	Core	Green
23[161]	11[167]	CACTGAGTCATTTTCCACCCTAGCGGGGGGAACCT	Core	Green
5[126]	1[125]	CAGGGCGACGCTGCGCGTAACAGAAAGGAGCCCCCTTGGGGT	Core	Green
0[125]	16[119]	CAGGGCGTTAAAGAGAGATAGGGTTGAGATCCTGTTTGATGGAGCAAGC	Core	Green
33[168]	15[174]	CAGTGAAAACCGGATTATTTT	Core	Green
13[140]	35[146]	CATCGGGTAATTTAGCACTCAGGTGCCTCTGCATT	Core	Green
20[146]	25[139]	CCAGAATAGGGATTGGCCTTGATATTCATACCGTTTGTAGCATATCGGC	Core	Green
28[132]	11[132]	CGAGCGTACTTCTGGAAATAAGCCCGTA	Core	Green
24[132]	20[126]	CGCTCATCTCAAACATACTTCATCACGCAGTGTTT	Core	Green
12[167]	35[167]	CTGAGCAATTCGCCTTATCCGGCCGTTTTATTCATAACGGTG	Core	Green
30[174]	13[174]	CTTGAGACCTCCCGAGAAGGCTGATTGC	Core	Green
25[140]	26[140]	CTTGCTGTGCAACAAACCGCCAATCAAT	Core	Green
29[140]	33[146]	GAAGGGTCTTACCAATATCCCCATTGTGGTAAAGC	Core	Green
6[139]	20[147]	GACGAGCCGATTAACCTGAGAAAATTAACCAGGAACCCATGTCGGTACG	Core	Green
19[140]	34[140]	GCAAAATGCCTGGCCCTGAGAAGCTGATTGCCCTTAATCGGC	Core	Green
9[147]	6[140]	GCGTCTCGACGATTTTAGACAACAACTAAAGCTTT	Core	Green
0[153]	3[160]	GCTTTGATGCAGGGAGTTAAATATTCGGTCGCTGACGGGCGCTAATTTC	Core	Green
16[118]	32[112]	GGTCCACTTCACCAGTGAGACAGGCGGTTTGCGTATTTCCAGTAATTGC	Core	Green
4[160]	8[161]	GGTGAGGAATAATAGAAAGGAGGTGTCGTCTTTCCGTTGAGG	Core	Green
11[133]	24[133]	TAAACAGTGATGATCCACCGGGGAAAAA	Core	Green
35[168]	21[174]	TACAGACAGGGAACGCTCCATAGATTTGTTTGACCCAACCTAGTTTCCA	Core	Green
23[119]	12[119]	TCTGTCCTTTGATTGCAGTCTTGTACTGTAACAGTAGAAATT	Core	Green
11[175]	24[168]	TGAAACAGAAGGATGGATAAGGGAATAGGTGTATC	Core	Green
28[174]	11[174]	TGCACCCTGAAACACAATTACATTATTC	Core	Green
3[161]	18[161]	TTAAACAACCGATAGGCCGCTCAACGGCTGAGGAAAAACGAA	Core	Green
26[174]	8[168]	TTACCTTACCAGGCTAGGATTCAGAGCCACAGGAG	Core	Green
20[125]	25[118]	TTATAATGCTAAACATAAATCCTCATTAGGAAAGCAGTAATACCTGAGT	Core	Green
30[132]	13[132]	TTATGCGGAACAAGGTAGAAAAACAGTA	Core	Green
12[153]	10[147]	AAATTATCCCCTGCCTATTTCTTGCCACCCATACA	Core	Green
14[153]	12[154]	AAGAACCAGAAACAATAACGGAATATCA	Core	Green
24[153]	9[146]	CAGCCATGTAATATAGCAAGCCCAATGTCCAGTAA	Core	Green

26[153]	24[154]	GTACATATCCCTCAGAGCCTTTTACCGC	Core	Green
33[147]	31[153]	CTCGTAACAAAGCTATTTCAACTTTAATATCGCGA	Core	Green
21[175]	3[174]	TTAAACGCGGAACGAGGGTAGTTTGCGGGATCGTCATCGCCCACGCATAGCTTGAT	Core	Green
3[175]	23[181]	ACCGATAGGTTTATAAAATCTAGTTTCAAGTAAATATAGTTAACCAGTA	Core	Green
8[181]	25[188]	AGCATTGACCACCCCAGAGCCTAGTACCGCCACCC	Core	Green
0[139]	0[140]	GAAAAACCGTCTATCGAGGTGCCGTAAAGCACTCTGGAGGGC	Core	Green
2[146]	3[139]	GGAAATCGGAACCCTAAAGGGAAGGGAAGAAAGCG	Core	Green
17[126]	21[139]	GGGCAACGAGTTGCTGGTTCCATAGCCCACGTGGACTCCAAC	Core	Green
34[160]	34[161]	ACCAACTTTGAATGCAGTGTCGAAATCCGCGACCTCGAACTG	Core	Green
23[182]	8[189]	CAAACTACCACCCTTCAGAGCCGCCACCCCAGAGC	Core	Green
21[140]	19[139]	GTCAAACTATAAATCAAAAGAGAAATCG	Core	Green
35[147]	15[153]	AAAGAGGACAGATGTACCCAAATCAAGGTCGAGAA	Core	Green
8[160]	25[174]	CAGGTCAAGAACCGCAGGGATCCAGAACAAACTCAGGAGGTT	Core	Green
10[146]	12[140]	TGGCTTTTTAATGCTTGCACG	Core	Green
24[167]	33[167]	ACCGTTAGCTCAGTTTTTAATAAGATGAAGCTACATAGCGAATGGTTTAGCTCATT	Core	Green
55[112]	60[107]	AATATAACTAATATCAGAGAGAGCAGATTTCCT	Core	Green
45[161]	51[167]	AATGCAATACTTTTAATTAAGCGCGAGCTTAGTTTTAAAGTATTGCGGA	Core	Green
70[139]	56[133]	ACCGAGCTCCTCACTTAGTAT	Core	Green
66[139]	41[146]	AGCCAGCTGTATAAGCAAATACCCCAAAGGGAATT	Core	Green
49[154]	46[154]	AGTAGATTGAAAAGGTGGCATCAAAGAATTAGCAAGCGGGAG	Core	Green
70[181]	56[175]	ATACCACAGCAACATAAGAAT	Core	Green
50[174]	47[174]	ATGCAACGACCATTAGATACAATTTTCATTTGGGGCAATAAA	Core	Green
65[119]	40[119]	CAGTATCGGCCTATTTTCATCAACATTAAAATACCTCAATAG	Core	Green
54[132]	54[133]	CCCTGGCCGGGTCACGGTGCCGGGGGGTAATAGTAGTCTTTA	Core	Green
39[140]	36[133]	CGGAACTGAACGTTTTTGCGGTTGAACAAAGTCAG	Core	Green
43[77]	47[83]	CTGCGCACGATTAAGTTGGGTTTTTCCCAGTCACGGCCATGTTTCTCCG	Core	Green
60[125]	70[107]	GAATTATAACAGCCAGACGACCGTTGGGCGTAATCATGGTCATAGCT	Core	Green
52[132]	52[133]	GAGTACCGCAAGAAGCGGTATGAACTATTATAGTCGATTAGA	Core	Green
59[175]	41[188]	GAGTCAAATCTAAACATTTGAGCAGCACTTACCATTAGCAAG	Core	Green
50[111]	47[111]	GCAAACGTTTAGTGATGAAGGACAGCGGATCAAACGGAAAAA	Core	Green
47[175]	43[174]	GCCTCAGTTATGACCCTGTAATGCCTGATCACCATGAGTCTG	Core	Green
47[91]	43[90]	GGGATAGGCGAAACGTACAGCACGTTGTTGCAAGGACTGTTG	Core	Green
71[175]	36[175]	GGTAGAATAACATAATACCGACGCGAGA	Core	Green
48[132]	48[133]	GTAGTAGCATTAAAGTAAAGTTAAACGTTGATTCCACTAATA	Core	Green
64[160]	41[167]	TGAACCTGAGGTGAATTCGCGTGTAAACGTTAATACCCGGTTCACCAGT	Core	Green
42[90]	39[83]	TGGTGCCGTAACCGTCTCCGTGGGAACATCTTTAAGGGCGACTTAGCAA	Core	Green
67[105]	55[111]	TTACCTGCGATCCAGCGTGCCTGTTCTTAAATTCTTACCAGTATAAGAG	Core	Green
57[133]	38[133]	TTATCCCCACCAGATTATCATATTAATT	Core	Green
66[146]	43[139]	TTCCTGTCAGAAGAGCTAAGTTTATTTTGCCATTTAACAGGAGAGAGAT	Core	Green
55[91]	38[91]	TTTTCGAACAAGAATTGAGTTAAGCCCTTTTTAAGAATAATA	Core	Green
55[140]	69[132]	ACCCTCAAGCCTGTAGTTGAGAAAAGAAGTTTTGCCAGAGTGGTGAGCC	Core	Green
53[148]	67[153]	AGCGGAAAATCAAAAATCAGAAATGTTTAGACTG	Core	Green
51[147]	53[147]	ATAAGAGCTCCAACAGGTCAGAGAAGCAA	Core	Green
38[132]	61[139]	TTAAAAGACGCAAAGCGTTTTTTATTTACATTGGC	Core	Green
61[140]	70[140]	AGATTAACGTCAGAAAACATAAAGAAACAATCCAAATTAACTTTAATAACCCGGGT	Core	Green
41[147]	63[139]	AGAGCCACAAGTTTTAAAACACAAATATCACACCAGTCACACCCACCAG	Core	Green

56[132]	55[139]	CATATGCACAAAAGGTAAAAC	Core	Green
64[181]	42[182]	AAATCTAACACCGCATAGGAAAAAATTCGCATTAATAGCATG	Core	Green
68[153]	56[154]	CTTTTGCGACAAAAGGAATTATAATTAC	Core	Green
66[104]	37[104]	ACAACCCGCCCTAATTACCAGTACATAAGAAACGCAAAAGTA	Core	Green
42[111]	66[105]	CCAGCTTTGAGGGGGCGAGTA	Core	Green
66[167]	37[167]	AAAAATAGGCGGTCAGTAGCGGTCAATACTCGTATAACTATA	Core	Green
70[167]	71[153]	TAATGCAGATACATAACGCTCAACGAAC	Core	Green
62[167]	71[167]	TAGGAGCTAAGACGTTTTTAATTTGTTTGGAAGCGACAACAT	Core	Green
67[84]	69[90]	TTAACGGCGCGCCTACCGGGG	Core	Green
68[97]	54[91]	GTCACTGCATCAGATGGGTAA	Core	Green
53[105]	67[104]	CGCCGGGCCTGCGGCTGGTAATGCCGGG	Core	Green
48[104]	51[104]	TTAAATTGGCGGCCCGGTCCGTCAGCGT	Core	Green
68[160]	53[160]	CGAGAGGGATAGCGTGACCATTTGCATC	Core	Green
67[168]	71[174]	CTGCGGAAAAAACCTAGTAAGATTCAACTATTACA	Core	Green
39[119]	39[111]	AAAAGAATTTTACCAGAAGGAAACCGAGAGGTGGC	Core	Green
57[175]	38[175]	AAAATGATCTGAGAGCTGATGAAACAAT	Core	Green
64[104]	65[97]	AACAGAGATAGAACACTGATAGTCGGATTGCATCT	Core	Green
39[112]	43[125]	AACATATAAAATTCTTATCACACTCCAGCTCTTCGCTGTAGC	Core	Green
52[160]	49[153]	AAGCAAAGTCATTTCGGTGTCTGGAAGTGCGAACG	Core	Green
46[153]	66[147]	AAGCCTTCCTCATAACCGTTCGGCTATCAGAAAAGTTTAAATTCTGGCC	Core	Green
64[125]	59[125]	AATAAAAAAATGGACATCGGCATTTTCC	Core	Green
38[83]	68[77]	ACCCAAACTTACCGAAGCCCATTTAGGCAGGGCTTCGGTCATGTGCACT	Core	Green
64[83]	65[76]	ACCTGAAAGCGTAACTATTAGAACGGCGTAGATGG	Core	Green
38[90]	43[97]	ACGGAATACGTAGAAAATACACGCCAAAAAATTATCCGCTTCGGAAGGG	Core	Green
44[125]	44[126]	AGAGGATTACGCCACTTTCAGAGGTGGAGCCGCTAATGCCGG	Core	Green
41[168]	61[167]	AGCACCACGTAATCAGTATTAAAGCATCACCTTGCCTGGTCA	Core	Green
37[119]	68[119]	CAAAGTGTTGAGCGAGTACCGGTTATACCGCGTCCCCCCCTG	Core	Green
68[118]	50[112]	CATCAGACAGCCAGCTGTTGCCGCGGTTTGCCAACGTCTGGTCGTTCCG	Core	Green
68[181]	50[175]	CGACGATATCGTCAAAACAGTGAAGCCCCTTCAAAGAGCTTATTTAAAT	Core	Green
43[98]	47[104]	CGATCGGAAAGGGGGATGTGCAAAACGACGGCCAGACAATCGCTCTCAC	Core	Green
43[140]	45[139]	CTACAAATAGCTGATAAATTAAAAATTT	Core	Green
61[107]	65[118]	CTCAATCGTCTGGGGACATCCATTAAAATGTGAACGACGA	Core	Green
59[154]	61[160]	CTTAGATACTAACAACTAAAGCCCTCAATCAATAT	Core	Green
46[125]	46[126]	GAACGGAAGACATCCAATAAATCATACAACGCAAGGACACGG	Core	Green
41[126]	64[126]	GACTTGAGTCACAAGAACGAAGACCAGT	Core	Green
47[112]	43[111]	GAGACGCTAACCTCACCGGAATGCCAAGGCTGGCGTGCGGGC	Core	Green
41[154]	45[160]	GCAAAATGATAATCAGGTCATTGCCTGACAATATGATATTCATATTTTA	Core	Green
48[83]	51[83]	GCCAGCATCCCGTACGCTGGCACGTGCC	Core	Green
65[98]	64[105]	GCCAGTTTCCGGCATCATTAAAGGTGAAATATGGTAACATCGTCTGGCC	Core	Green
65[77]	64[84]	GCGCATCGGAAACCGTAAATATTGACGGGACAAAATGCGCGACCTTCTG	Core	Green
51[84]	53[83]	GGACTTGATCCCACGCAACCAGTCATTG	Core	Green
50[90]	47[90]	GTCTCGTAAAAAAGCCGCACATCTGCTCATTTGCCTGGTGAA	Core	Green
37[154]	40[154]	GTTATATTAAATCCTTTGCCCTTTAGATTAGAGCCACAGAAT	Core	Green
69[91]	55[90]	GTTTCTGCAACGCTCAACAGTAGAGGCA	Core	Green
71[154]	37[153]	TAACGGACATTAGAACCTAAAATATTTTAGTTAATAAGTTGG	Core	Green
43[126]	41[125]	TATTTTTAGCAGGAAGATCGCCGTCACC	Core	Green

42[181]	45[188]	TCAATCAGAGCAAACAAGAGACGGAGACAGTCAAAGTAATGTGTAGGTA	Core	Green
59[133]	39[139]	TCCTTGACTGTAGCGACACCA	Core	Green
38[174]	61[181]	TCGACAAGATAATAATATCTTGTTGGCAAATCAAC	Core	Green
50[125]	50[126]	TGATTGCCAGCAGCAACCTTTAATTGCTCATATAACAGATGC	Core	Green
45[140]	51[146]	TTAGAACTATTTCAGGCAAGGCAATTCTCAATTCTTTCATTCCCTTTTG	Core	Green
53[91]	50[91]	TTTCGCATGGTGCCTAGAACGTTTTTTC	Core	Green
42[174]	66[168]	TATGTACTTTTGTTCGCCATC	Core	Green
56[174]	76[168]	AAACACCGTTTGAAAAAACAGAACGTCATTTACACCACAACCAAA	Fluorophore OH	Cyan
36[174]	74[168]	AAACTTTTGTAAATGACTACCCTGAGAATTTACACCACAACCAAA	Fluorophore OH	Cyan
15[133]	77[139]	AAGTACCCGAGCATAAAAATAACGCTAATTTACACCACAACCAAA	Fluorophore OH	Cyan
57[154]	74[147]	ACGATTTCCTCCGGCTTAGCAGCGATAGTTTACACCACAACCAAA	Fluorophore OH	Cyan
36[132]	74[126]	AGGGTAAAGTAACAAGGAGCGCTTAGAATTTACACCACAACCAAA	Fluorophore OH	Cyan
15[154]	77[160]	CAAGCAAGTATTCTGGCGTTTATTTTATTTTACACCACAACCAAA	Fluorophore OH	Cyan
15[175]	77[181]	CATCGTACAGATATACTTGCGGCTATTTTTTACACCACAACCAAA	Fluorophore OH	Cyan
13[133]	75[139]	CCTTTTATAAAACAAATAATGATATGTGTTTACACCACAACCAAA	Fluorophore OH	Cyan
13[175]	75[181]	TTTGAATTTCATTTAACATCAATTTGAATTTACACCACAACCAAA	Fluorophore OH	Cyan
28[153]	75[160]	CCTGAATTAGAACCTACCAAGGGAAACATTTACACCACAACCAAA	Fluorophore OH	Cyan
70[132]	76[126]	TCGAATTAAGAAAAATCTACGGAGTAATTCTGTCCATATTATTTTACACCACAACCAAA	Fluorophore OH	Cyan
1[182]	19[181]	ACCCTCAACAGCATGGTAAAACACTACGAAGGCACCCCAGCG	Neighbor Staples	Red
19[182]	34[182]	ATTATACACAAAGTACAACGGGTTACTTAGCCGGAGACGGTC	Neighbor Staples	Red
5[189]	2[182]	AAAGGCTCCTTTAATTGTATCGTTGCGCAACAACC	Neighbor Staples	Red
7[189]	5[188]	CTGTATGAAACAACTTTCAACCCAAAAA	Neighbor Staples	Red
25[189]	8[182]	TCAGAACAGAACCGCAACGCCCACAGACAGCCCTCGAATTTTCGCCGCC	Neighbor Staples	Red
27[107]	24[107]	AATCAAAATCACCCTACATTTTGA	Neighbor Staples	Red
31[107]	28[107]	TCAACAATAGATGAGCCTAATTTG	Neighbor Staples	Red
29[107]	26[107]	TCCTGATTGTTTCTTCTGTAAATC	Neighbor Staples	Red
33[107]	30[107]	TTCCACACAACAGAACTGGCTCAT	Neighbor Staples	Red
41[189]	36[182]	GCCGGAATGAAACCATCGATAGGATTTAACTTTACCAAATCCCAAAGAA	Neighbor Staples	Red
66[188]	64[182]	TTAACCACTGCAACAGTGCCACAGCAGCAAATGAA	Neighbor Staples	Red
57[182]	70[182]	AAATAGCGAGAGAAAGATTCATTTAGGA	Neighbor Staples	Red
45[189]	66[189]	AAGATTCGAAAGGCATCGATGGTAAAACATTTTTGTCATTTT	Neighbor Staples	Red
61[182]	60[182]	AGTTGAAGAAGGTTTAGTGAATCATAGG	Neighbor Staples	Red
55[182]	68[182]	CCGTGTGCGTTAAACTATCATTACCAGA	Neighbor Staples	Red
57[107]	37[118]	AGTTACAAAATACATCATAAGCCGAA	Neighbor Staples	Red
59[107]	62[107]	CGCTATTAATTAATTTTCGGTCAT	Neighbor Staples	Red

71[107]	58[107]	TACCAGTCAGGAGACAATAAACAA	Neighbor Staples	Red
53[84]	80[77]	CAGGCGCAGGTTTCTTTGCTCCAAATCG TTTCTCTGGTTAACGTGT	Biotin OH	Pink
51[105]	82[100]	GGTGCTGGGCAGCACCGTCGGCTCAATC TTTCTCTGGTTAACGTGT	Biotin OH	Pink
51[168]	82[161]	TGGCTTAGCGAACCAGACCGGAAAAAGA TTTCTCTGGTTAACGTGT	Biotin OH	Pink
53[168]	80[161]	TTAAGAGTCAGAAAACGAGAATCCAATA TTTCTCTGGTTAACGTGT	Biotin OH	Pink
36[104]	81[101]	ATAACCCGCCAGTAATAAAGCCCAGCACGCGCAGT TTTCTCTGGTTAACGTGT	Biotin OH	Pink
36[167]	81[165]	TTCAAATTTTAATGGGAATCACGAGGCAAAAATAG TTTCTCTGGTTAACGTGT	Biotin OH	Pink

Supplementary Table 4. List of ssDNA staple sequences for polymerization of the top nano-brick. Nano-brick polymerization staples are shown on the left edge of the brick cadnano design (Supplementary Fig. 4).

Start	End	Sequence	Type	Color
33[21]	32[21]	GGTATAAAAGATTAAGAGGAAAAAGCGGATTGCATCATAAAC	Polymerization Staples	Red
41[28]	40[28]	ACGCGAATTTTTAGAACCCTCTCAACGCAAGGATAAAAGAAA	Polymerization Staples	Red
17[28]	16[28]	ACCGTAGGAGCCTTTAATTGTAAAAAAAGGCTCCAAACACCG	Polymerization Staples	Red
35[21]	34[21]	GCGCCCATTTTTGCGGATGGCCCTTTTGATAAGAGGTTGTTT	Polymerization Staples	Red
31[21]	30[21]	GGTATCCAATACTGCGGAATCTTAGACTGGATAGCGTTCTAA	Polymerization Staples	Red
13[21]	12[21]	AGAGCCCCAATAGGAACCCATTTTCAGGGATAGCAAGCACCA	Polymerization Staples	Red
63[21]	62[21]	ACCAGCGCGTACTATGGTTGCATGCGCCGCTACAGGGTCACA	Polymerization Staples	Red
45[28]	44[28]	AATCAGAAAAGCCCCAAAAACCCCCGGTTGATAATCAATATA	Polymerization Staples	Red
43[28]	42[28]	AGTGAGAGAGGGTAGCTATTTTGATAAATTAATGCCGATTTA	Polymerization Staples	Red
61[21]	60[21]	GTCTTGCTTGACGGGGAAAGCGAGCCCCCGATTTAGATAATG	Polymerization Staples	Red
29[21]	28[21]	TCTTAGGAATTACGAGGCATAATACATAACGCCAAAACCAAC	Polymerization Staples	Red
39[28]	38[28]	CGGAATAAATCATACAGGCAATAGCATTAACATCCAATCATA	Polymerization Staples	Red
37[21]	36[21]	TGTAATAGATTTAGTTTGACCCAATTCTGCGAACGAGTTTAG	Polymerization Staples	Red
47[28]	46[28]	AATTATCGCGTCTGGCCTTCCCGCCATCAAAAATAATTTCAT	Polymerization Staples	Red
19[28]	18[28]	CACAATGCAGGGAGTTAAAGGTTCGGTCGCTGAGGCTTCAAT	Polymerization Staples	Red
15[28]	14[28]	TAATCTTAGTAAATGAATTTTGTCGTCTTTCCAGACGAGTAG	Polymerization Staples	Red
52[34]	53[34]	AGCTCGCGGAACAAAGAAACCGTAACATTATCATTTTGAATT	Polymerization Staples	Red
48[34]	49[34]	ATCGTAGGTTTAACGTCAGATAATTGCGTAGATTTTCAACCG	Polymerization Staples	Red
26[34]	27[34]	ATTATTAGCAGCCTTTACAGAACGTCAAAAATGAAAAACCAG	Polymerization Staples	Red
50[34]	51[34]	CGGGCGGATTATACTTCTGAAATAATCCTGATTGTTTCTCTT	Polymerization Staples	Red
56[34]	57[34]	GCAGCGTTGAAAGGAATTGAGAGTTGGCAAATCAACAAAGCG	Polymerization Staples	Red
22[34]	23[34]	GCGACCTTTTTAAGAAAAGTACTATCTTACCGAAGCCCTGCT	Polymerization Staples	Red
58[34]	59[34]	GTGGAGCTGAGAGCCAGCAGCCCTGCAACAGTGCCACCTCCA	Polymerization Staples	Red
24[34]	25[34]	TCATTCTAATATCAGAGAGATAGAGGGTAATTGAGCGACCCA	Polymerization Staples	Red
54[34]	55[34]	TCCAGAGTATTAGACTTTACACATTTGAGGATTTAGATCGGG	Polymerization Staples	Red

Supplementary Table 5. List of ssDNA staple sequences for folding the bottom nano-brick. The start and end columns denote the helix[base] positions at the 5' and 3' ends of the DNA staple strands in the cadnano design (Supplementary Fig. 4). Color is also with respect cadnano. Note the designs for the top and bottom nano-bricks are similar except the start position of the scaffold is different.

Start	End	Sequence	Type	Color
21[112]	49[104]	AAAAGAAATAAAAGTGCGTAGCTGATTGCTTTGAATACCATAAGGCAAA	Core	Green
44[62]	47[55]	AAAAGCCACAGCTTGCAGGGAGTTAAAGGAAAGACGGCCTTCGCGAGTA	Core	Green
29[56]	60[56]	AAACCAACCAAAAGGCGCTAGTTTAGAG	Core	Green
23[70]	49[66]	AAAGAGGTGTCGAATTTCCCAGGCGATCGGTGCGGCCAG	Core	Green
59[63]	64[63]	AAATCAACCCCCGAGGCGCTGCGCCGCTAACAGGAGTTTTTA	Core	Green
41[91]	45[97]	AAATCACCATCAATGATTTTTTCACGTTACAACCAGCATGTCAAATCAG	Core	Green
38[62]	33[62]	AAATCATTTCATTTAACGAGTAAATATGAGAGGTCGAACCAG	Core	Green
38[72]	43[76]	AACCGGTTGTACGCAAGTGAGAAATAAATTAAAGAGAA	Core	Green
13[49]	45[55]	AACGCCTTGAATTTAGTTTCATTAATTGTTCTTAACCAAAAATAAACGT	Core	Green
14[86]	35[80]	AAGACATTATACTAATAGTAGTAGAAAGGTGCAGTTGATGTC	Core	Green
60[76]	55[76]	AAGGGAGGTTTTTTTATTAAACCTTATACTGAGAGGCGCCAG	Core	Green
48[132]	43[132]	AATAACGTATTCATTTAATTACAATATATTAGAATGAATTTA	Core	Green
22[125]	56[119]	AATAATAGAAGCCCATGAAATTGAGCGCTAGAGCC	Core	Green
19[126]	40[126]	AATCAATGATTGAGGTCACCGCATTAGCCGAGAAATTTGAAA	Core	Green
37[119]	32[119]	AATTTAGTAAAGTACCTGTTTCGAGCATATTAAACAATCATT	Core	Green
47[56]	51[62]	ACAACCCGTAACCGACTCCAGGCCTCTTGGTAACG	Core	Green
8[117]	9[118]	ACAGGAGTGTACAGTAAGCGTCATACA	Core	Green
62[62]	25[52]	ACAGGGCGCGTACTGGAGCGGGAATTACGGAACAATCAGGACGACG	Core	Green
32[90]	64[98]	ACTATTAGTCAGGATTCTTTCCTTATCACAAGCAAAACTATCTGAAATG	Core	Green
23[56]	57[69]	ACTGACCTTACCCAAACACCAGCAAGCGAAATCGGCAAAATC	Core	Green
54[76]	23[69]	ACTGCCCGCTTTCCAACATACATATTCAAACTTTG	Core	Green
25[137]	22[126]	AGAAAGAGCAAAGAAACTGATTATCAGATGAGATTATAGAAACGC	Core	Green
25[53]	22[45]	AGAAATCAACTCACAATCGTAATCATGGTCAAGGCGATACTT	Core	Green
62[139]	25[136]	AGAACCCTGATAGCCAAAATATTTACAGGACGGGAAGAG	Core	Green
57[84]	25[97]	AGAATAGGAACAAGTTGAGATTTAGGGAGAATTACGTTTAAT	Core	Green
11[133]	37[132]	AGACGATGCCACCCTCAGAGCCCGGAACCAAAAGGGCAGAGG	Core	Green
34[90]	11[97]	AGAGTACAAGTTTCATAACATGTTCAGCATAATCGGCTGTAGCCCAGAG	Core	Green
11[81]	62[73]	AGATCAGTACATCAAAATAGTAAATAAAGGGCGCCGCGCTTA	Core	Green
12[58]	63[62]	AGCCGCCACCCCGTCGAAGAAAACTAGCGTCGGAGCTA	Core	Green
48[90]	42[84]	AGGTCACAACGGCGACCAATATTTTGTTAATCATAACGGTAAACCGTTC	Core	Green
65[63]	10[70]	AGTAATAACATCACAAGCAAAACCATAACAGGCGG	Core	Green
64[44]	10[49]	AGTTGTAGCAATACTTCAAAGATTACAGTTCGAGGGTT	Core	Green
10[69]	13[73]	ATAAGTGCTCAGAATTTGATACAACTAAAGTACGGTTCCCAAACAG	Core	Green
13[98]	37[90]	ATCAAAACAGAACCACCACCTCAGAGCCTAGCGTACATATAAGCATCAA	Core	Green
62[72]	24[70]	ATGGCAAGTGGCAGATAGAAAGATAGAACTGTAGTAAAGAACCGG	Core	Green
45[77]	22[77]	ATTAAATGGAACGCACGGCTAAGGAAGTCCCCAGCTAAATTG	Core	Green
8[97]	6[84]	ATTAAGAACCTATTCGTATAAACAGTTA	Core	Green
11[109]	14[112]	ATTCCAATCATAATGCAGAACGCGATTCTGTAACCAGACCCCCTT	Core	Green
31[119]	62[119]	ATTCTAATAAATCAAGTCACACTGAAAG	Core	Green

62[97]	30[91]	CAAACCACCACACCATTTTAGACAGGTTCCCAGAG	Core	Green
28[90]	61[101]	CACATTCAAGAAGTTTTGCAGCTACAATATAAGAAATTT	Core	Green
12[69]	38[73]	CAGGGATTCCACAGTTCTGCGGGGGCGCGAGCTGACATT	Core	Green
45[119]	21[125]	CATAAATCATTTAATTGTCACGCAACATCTGGCAT	Core	Green
46[76]	50[80]	CATCAAATCTCCGTGGGAACAGTTGGTGCCGCTTCTGGG	Core	Green
24[97]	51[90]	CATCAAGAAGCCTGGCAGGTCGACTCTACGTTGTA	Core	Green
17[105]	40[91]	CATTAAACACCAATCCAATCGTAAATAAGGCTTTTGCGGGAG	Core	Green
37[133]	42[136]	CATTTTCCTTACCAATTACTATTAATGGACTTTTTGGTT	Core	Green
32[111]	12[112]	CCAATAGTTCCAAGAACGGGTGTAGAAAGACAGGACTCAGAG	Core	Green
59[42]	64[45]	CCACTACGGGAAAGAGCGAAAATGGTTGTTAGAATACCG	Core	Green
15[105]	39[97]	CCATCGAATTAGCGTTAAATAGTAGGGCTTAATTGAGAATCTGACCCTG	Core	Green
13[74]	45[76]	CCCGTCTTTCGAAAGGAAAAAGGCATAGTTGCGGTTGAAATTCGC	Core	Green
60[139]	55[136]	CCGAACGCCAGCAGACCTCAAGAATTGAAACAACTACTT	Core	Green
12[111]	37[118]	CCGCCACTCACCGGCCAGACGCAACGCCAACATGT	Core	Green
40[146]	14[140]	CCTAAATGAAAAAGGCGTTTT	Core	Green
44[125]	46[115]	CCTTGAATATCACCGGAGGGAAGGTAAAGTTTATTCAAT	Core	Green
47[98]	19[104]	CGAGTTACAAAATCATTTGAATTACCCAACACCAC	Core	Green
42[146]	15[146]	CGGCTTACAAATATAGTAGCAATCAAGT	Core	Green
62[118]	26[105]	CGTAAGAATTAGTCTTATCCCTCAAAAACCCTGAACAAAGAA	Core	Green
59[126]	65[132]	CTGAGAGAACCACCCGCGAACTTCTGACCGACCAGAATACCTAACAATA	Core	Green
65[112]	11[108]	CTGGTAAACCGCGCATCCGGTTGGAAAGCGCAGTCCAGC	Core	Green
49[67]	41[66]	CTTAAACGGGGAACGAGGAGGCTTGATACCGTCCAAAAAAAA	Core	Green
60[55]	54[49]	CTTGACGGTGAACCACGTCAAGGTTCCGGTCCACGGAGAGGCAAACCTG	Core	Green
21[70]	47[66]	CTTTGACTTCCATTTCCGGCATAGATGGGCGCATCGTCG	Core	Green
45[98]	17[104]	CTTTTTTTAATGGACGATAGCTTAGATATCTTATT	Core	Green
23[133]	20[126]	GAAAAGTAACCGAGCTTCTGAATTATTTGCACGTAGGTTTAAAAAGGTG	Core	Green
64[97]	32[91]	GAAACGGTACGCCAAGTAGAAGAACTCAATCCCTG	Core	Green
12[48]	38[52]	GAACCCAAAACTACGTTTGACTGTTTAGCTATATTACAG	Core	Green
23[49]	20[42]	GAACCGACCATGTTTAAGTTGCGCTATTACGCCAGCTCAGGACCACTAC	Core	Green
31[126]	34[133]	GAACGCGTCGTAGGCAAGTACCGCACTCCCCATCC	Core	Green
58[69]	31[69]	GAACGTGACAGGTACATAACGAATAGCGGACTGGAGAGAATG	Core	Green
53[70]	21[69]	GAGCCGGCCCGGGTACCGAGCCCAGGGTATCCGCGCACTCAT	Core	Green
47[67]	41[59]	GATAATAATTTTGTTAATAATCAGAGCAAACATGCCGGAGAGGGTAAGA	Core	Green
10[48]	36[49]	GATATAAAGGAGGTCGGATGGGCTCAACATGTTTTAGATTTA	Core	Green
63[74]	60[77]	GATATGTTTAAGAGGCTTTTGCAAAACTAATTAGCGGTAACCCTA	Core	Green
38[51]	43[62]	GCACCTCAGAGAACCCTGTAGGTAAGCTATTGCCTGAGAGTCTGG	Core	Green
32[62]	29[55]	GCATCAATTTGATTTAATCAGTGAGGCCCAGAGCGCAATACTACGATAA	Core	Green
49[105]	21[111]	GCGCCATAAACGCAAAGACTACGAAACAAAGTACC	Core	Green
55[42]	26[42]	GCGCGGGCTGGTTTTTGCCCTGTTGGGA	Core	Green
32[69]	12[70]	GCGGATTACCGGAAGCAAACTTGCTCCTCCGCCACTCATTTT	Core	Green
54[104]	54[105]	GCTAACTTGAGACGGGTACATTTGAGGAAAGTTTGAGAGTGA	Core	Green
31[42]	12[49]	GCTTTAAAAGAGGATCGAGCTTCAAAGCATTTTTGTTAGTACCCAATAG	Core	Green
19[105]	47[97]	GGAATAATATTGACGGAAAGCCCACGCATTTGAGGTTTTTTAGATTGAC	Core	Green
41[67]	12[59]	GGGGATAAAAATTTTTAGCATAAATAGTAAAGTAGCATAGCA	Core	Green
30[90]	11[90]	GGGGTAAATCAGGTCTTTACATTGCGGGGTTTTGCACCGCCA	Core	Green
42[135]	18[140]	GGGTCAAAATCATAGGTTAATTAACCATTTGGGCGACA	Core	Green

60[107]	65[111]	GGTAATGGCTATACGTGTTGGCAGAATCGTCGGCCTTG	Core	Green
6[117]	7[117]	GTGCCTTGAGTAACAGTGCCATTCTGAAACTGGTAATAAG	Core	Green
39[116]	43[125]	TAACGTGTGACAAGACAGTAAATGGAAGAGTCAATAGT	Core	Green
39[98]	15[104]	TAATACGTTTGCCATCTTTGATGCGAATAATAAAA	Core	Green
45[56]	22[56]	TAATATTCGCGTCTAGCATCGTAAAATAACTAAAAACCTGCT	Core	Green
16[135]	19[146]	TACACTTGAGTTTTCCCTGTGAGTGAATAACTCAAGAATCATATG	Core	Green
23[91]	49[90]	TACAGACGTATCATAAACGACGGCTGCGCAACTGTTGGTGCC	Core	Green
55[137]	23[132]	TACCCCGAACGTTATTACGGAACAAGAAACATTTTTAA	Core	Green
40[125]	10[119]	TACCGACACACCGGGTCATAGGCCACCACACCACCGGTTGAGGCCAGAA	Core	Green
42[83]	14[87]	TAGCTGAGGCCGGATCCAAAAACAACTAAAGGAATTCTA	Core	Green
65[119]	30[115]	TATCCAGACATTTTGACGCTCATTCACCAGAT	Core	Green
42[96]	42[97]	TATTCATCGTAAAACTTAAGACGCTGACTGATGCAAATATGA	Core	Green
40[83]	11[80]	TATTTCAACCAAAATTTTGTCTCATAGTACCACCCCCTC	Core	Green
57[119]	30[126]	TCAACAGTTGAAAGATATCAAAGCAGCCAACAGCCGCTAACGGAAGCCT	Core	Green
15[126]	39[115]	TCAGTAGATTTTCGAATCATAGTATAAAGCCAACGCTCAACAAGAA	Core	Green
64[139]	29[139]	TCATGGATAATAAAAGGTTTTAGCGTCT	Core	Green
26[104]	60[108]	TCATTGTTTTTTTGGTTGTTCTATTAACACCGCCTCAGA	Core	Green
52[62]	25[69]	TCGAATTTCCACACAGTCGGGGGTTTGCGTATTGGAGTTGCAGAACGAG	Core	Green
43[77]	19[76]	TCGATGATGTACCCCGCCGACTATATTCGGTCGCTGGTAGCA	Core	Green
54[48]	23[48]	TCGTGCCTATCCGCGTAACAACATAAGG	Core	Green
58[111]	31[111]	TCTGGGTTTTAACGAATCCAATTTATCCTTGCTATAAGGCTT	Core	Green
57[42]	30[42]	TGATGGTAGGGCGAAACTAACGAGGCATCCAGACGGCGGAAT	Core	Green
35[81]	63[73]	TGGCTTTAATCCAACAGTAGTCAGTTGCCTGGAATCCTGAGAAGTGGCC	Core	Green
34[128]	16[136]	TTAATCAACAATAGATAGTACCGACGCCTCCCATCGGCCGACAGACCAT	Core	Green
65[133]	34[129]	TTACCGCATTTTCAAGGCGTTAAATCCTCATTAAAGCAGGTCTAAT	Core	Green
42[132]	15[125]	TTATATAACTATATAAGAACGAAGGCCGACCGTAA	Core	Green
18[139]	47[146]	TTCAACCAGAAAATAACAAAATTCAATTACCTGAG	Core	Green
37[91]	13[97]	TTCGCCATATTTAAACGACAATAAACTCACTCATA	Core	Green
46[114]	51[122]	TTCGCGCAGAGGCGAATGATTCGCATTTTCAAAACAGATTGT	Core	Green
41[60]	38[63]	TTCGGAGCCTGCGGAGTGAGAATACAGACGTGCTAAATATCCAAT	Core	Green
61[102]	28[91]	TTGGAGGCGGTCGCACTAAATCGGCACGCTGCGCGTATACAATAC	Core	Green
20[90]	40[84]	TTTCATGCAGAGGCTAACCGAAATGACAGAAAATCGACAGTCAAGCCTT	Core	Green
7[84]	8[98]	TTTCGGAGGCTGAGGGATTAGGATTAACCGTTCCATGAAAGT	Core	Green
50[111]	23[104]	AAATTCGCCATTCAGGCCAGTGCCAATAACCAACGGAGATTTCAGGCGC	Core	Green
22[44]	48[49]	AGCAGGCAAAAGAATACCGTAATGAGATCGCTGCATCT	Core	Green
23[105]	50[112]	ATAGGTATCTTACCACGGAATATCCTGAAATAAAG	Core	Green
56[97]	23[90]	ATTGCCCTCACCAGCACATTAATTGCGTAAGTGTAAGTAATCAACGGTG	Core	Green
60[118]	58[112]	GATAAAAGCAACAGATCAATA	Core	Green
56[111]	52[112]	GATAACATTTCAGAGGGTAATAGCAATAACATTATTCATCAA	Core	Green
57[105]	56[112]	GATCAGTTGGCAAAGTCAATA	Core	Green
26[83]	59[90]	GATTTTATCATCAGAGTCCACGGGGTCGAGGTGCC	Core	Green
48[48]	51[41]	GCCAGTTTGAGGGGTATCGGCCTGGCGATGCTGCA	Core	Green
52[111]	56[98]	TAGCTTGCATGCCTGGGTGCCTAATGTAGCCTGGCTGACCTTTTCAACTACAGCTG	Core	Green
51[123]	56[126]	TTGTGGCAATCATTTTGATTTTAATTTAGAAGTATTAGAATAGAT	Core	Green
55[84]	26[84]	TTTCTTTTTCACCGTGAGATGCTTATGC	Core	Green
50[79]	55[83]	AAGGTCACGAGAGGATCAAGCATATGCGCTCGGTGGTTCAATCAGCAGCGAAT	Fluorophore	Yellow

			Core OH	
11[98]	62[98]	CCGCCGCTCTGAATGATATAGTTTGCACATTTACAGCACAGACAATCAGCAGCGAAT	Fluorophore Core OH	Yellow
22[90]	48[91]	CGCCTGAGATTATACCAAGCGAAGACTTGGAAACCATGGGATCAATCAGCAGCGAAT	Fluorophore Core OH	Yellow
21[126]	48[133]	GATTAAGACTCCTTCATACATCGTCAGAGAGAAACCAATCAGCAGCGAAT	Fluorophore Core OH	Yellow
15[112]	45[118]	TAGCAGCGAAACGTGGTGAATAACATAGAACAGTACAATCAGCAGCGAAT	Fluorophore Core OH	Yellow
26[41]	59[41]	AGAAAAATAAAACGAAAACCGCGATGGCCAATCAGCAGCGAAT	Fluorophore Neighbor OH	Orange
33[140]	64[140]	ATCGAGACGTTTTTCAGCCATAAAACGCCAATCAGCAGCGAAT	Fluorophore Neighbor OH	Orange
47[147]	42[147]	CAAAAGAACAAACACTTGCTTTCGCTATCTGAGAGTAACCTCCAATCAGCAGCGAAT	Fluorophore Neighbor OH	Orange
52[41]	55[41]	TAGCTGTAAATTGTAGCTGCAGGCCAACCAATCAGCAGCGAAT	Fluorophore Neighbor OH	Orange
29[140]	60[140]	TTCCAGACCAGTTACCTAAAAAAAAATACAATCAGCAGCGAAT	Fluorophore Neighbor OH	Orange
24[41]	57[41]	AGCTGCTATAAGGCGCCCCAGTCCTGTT	Neighbor	Black
13[140]	10[140]	CTCAGAGCAGAACCTGGCCTTAACAAAT	Neighbor	Black
20[41]	23[41]	GAAGGCAACGAAAGCGGAACGGGTCAAT	Neighbor	Black
37[140]	34[140]	GAGCCAGATATAAAAGTCCTGATAATAT	Neighbor	Black
19[147]	40[147]	GTTTACCACAAAAGGGAATTAAATCACCATTTTAGCTTCTGA	Neighbor	Black
31[140]	62[140]	TTAGCGATTGCGGGAGGGACACAGAGAT	Neighbor	Black
15[147]	38[140]	TTGCCTTCTGTAGCCCTGTTTGCGTTATACAAATT	Neighbor	Black
23[77]	68[69]	ACAGATGTTGACAATTGGGCTCCTGGCCAATCAAACTCTGGTTAACGTGT	Side Biotin OH	OH not shown
26[69]	66[57]	GCTCATTATACCAGCATTATTGACTCCAATCACCCCTCTGGTTAACGTGT	Side Biotin OH	OH not shown
59[91]	68[90]	GTAAAAGCAGTTTGCCCGAGATAGGGTTCTCTGGTTAACGTGT	Side Biotin OH	OH not shown
25[126]	66[122]	TAATATCGAATTAACTGAACATGAAAATACCCTCATGCCACGCTCTGGTTAACGTGT	Side Biotin OH	OH not shown
30[114]	67[122]	TAGTGAATCTTACCAACATATTATTTTAATGAGCAGAACTCTGGTTAACGTGT	Side Biotin OH	OH not shown

Supplementary Table 6. List of ssDNA staple sequence for polymerization of the bottom nano-brick. Nano-brick polymerization staples are shown on the left edge of the brick cadnano design (Supplementary Fig. 4).

Start	End	Sequence	Type	Color
39[28]	38[28]	AGTATAAATTAAGCAATAAAGAGGCAAAGAATTAGCACATAT	Polymerization Staples	red
33[21]	32[21]	ACAAGAATATCGCGTTTTAATAGCCCGAAAGACTTCACAAGC	Polymerization Staples	red
41[28]	40[28]	TTAATAATGCCTGAGTAATGTCATATATTTTAAATGCTTCAT	Polymerization Staples	red
43[28]	42[28]	ACTACAGGCTATCAGGTCATTTTTGAGAGATCTACAACTTTT	Polymerization Staples	red
37[21]	36[21]	TAATACAAATGGTCAATAACCCATTAGATACATTTCGAGAGA	Polymerization Staples	red
19[28]	18[28]	AGCGCCGTCACCCTCAGCAGCGCCGCTTTTGCGGGATCAAAG	Polymerization Staples	red
15[28]	14[28]	TAGCGCTAAACAACTTTCAACTCTGTATGGGATTTTGTCAGA	Polymerization Staples	red
61[21]	60[21]	CATCGGAAAGGAAGGGAAGAACCGGCGAACGTGGCGACCATT	Polymerization Staples	red
45[28]	44[28]	CTGTAGCAAATATTTAAATTGCAGGAAGATTGTATAAAATCG	Polymerization Staples	red
13[21]	12[21]	CCGCCGTTTCGTCACCAGTACTGTACCGTAACACTGAACCCT	Polymerization Staples	red
63[21]	62[21]	TTCTGTAACGTGCTTTCCTCGCTTTGACGAGCACGTAGCCAA	Polymerization Staples	red
47[28]	46[28]	AGATGTCAACATTAAATGTGACTGTAGCCAGCTTTCAATGAA	Polymerization Staples	red
29[21]	28[21]	GCCTATCATAACCCTCGTTTAAGTAAGAGCAACACTAATTTG	Polymerization Staples	red
35[21]	34[21]	AACAATGAATATAATGCTGTACTTAGAGCTTAATTGCGAAAA	Polymerization Staples	red
31[21]	30[21]	ACCTCTGAATCCCCCTCAAATCGTCATAAATATTCATCCGAC	Polymerization Staples	red
17[28]	16[28]	GAGCCTGCTTTCGAGGTGAATTATCGGTTTATCAGCTAGCAA	Polymerization Staples	red
50[34]	51[34]	AAGGGAACCTACCATATCAAAATAATGGAAGGGTTAGGGATG	Polymerization Staples	red
48[34]	49[34]	ACGACGTACCTTTTACATCGGTGAATATACAGTAACAGACAG	Polymerization Staples	red
22[34]	23[34]	AGGCGAAAGTTACCAGAAGGAAAGCAGATAGCCGAACCAGAC	Polymerization Staples	red
56[34]	57[34]	CAGGCTATCTTTAGGAGCACTGGAAGGTTATCTAAAAGAAAA	Polymerization Staples	red
24[34]	25[34]	CATTCAGTTAAGCCCAATAATTAACCCACAAGAATTGAGTGA	Polymerization Staples	red
26[34]	27[34]	TCTACACAGGGAAGCGCATTAAGAGAATAACATAAAAGTTAA	Polymerization Staples	red
58[34]	59[34]	TCTATAGCATCACCTTGCTGACAAATGAAAAATCTAACAGGG	Polymerization Staples	red
54[34]	55[34]	TTAATCGTATTAAATCCTTTGAAACAATTCGACAACTGAATC	Polymerization Staples	red
52[34]	53[34]	TTCCTATTATCATCATATTCCCACCAGAAGGAGCGGAGTGTG	Polymerization Staples	red

Supplementary Table 7. List of ssDNA staple sequences for attachment of the top and the bottom nano-brick to the nano-hinge. Polymerization staples connecting nano-brick to hinge arms are shown on the right edge of the nano-hinge cadnano design (Supplementary Fig. 6).

Nano-hinge to Top Nano-brick Polymerzation Staples				
Start	End	Sequence	Type	Color
30[202]	33[202]	TCCAGACCAGAACGAGTAGTATGCCCTGACGAGAAACTCGGG	Top Polymerization Staples	Yellow
14[202]	15[202]	TCATTCCAATAGCAAGCAAATGGAATCATTACCGCGCACCCA	Top Polymerization Staples	Yellow
10[202]	11[202]	GGGTACTGAGACTCCTCAAGATGAAAGTATTAAGAGGAAATA	Top Polymerization Staples	Yellow
24[202]	27[202]	ATCGTGATATAAGTATAGCCCTGCCGTCGAGAGGGTTAACCG	Top Polymerization Staples	Yellow
12[202]	13[202]	GCGACGCGCAGAGGCGAATTAACCAAGTTACAAAATCCTGCT	Top Polymerization Staples	Yellow
28[202]	31[202]	AGCTCTAAATCAAGATTAGTTGGAGGTTTTGAAGCCTGAATT	Top Polymerization Staples	Yellow
26[202]	29[202]	CGGGCTACATTTAACAATTTCAGAAAACAAAATTAATCTCTT	Top Polymerization Staples	Yellow
32[202]	35[202]	GCAGCTCATCAAGAGTAATCTATAGGCTGGCTGACCTAAGCG	Top Polymerization Staples	Yellow
9[196]	8[196]	AGAACTGCAGGGAGTTAAAGGTTCGGTCGCTGAGGCTCACCA	Top Polymerization Staples	Yellow
19[189]	16[189]	CAAGCGGAATTACGAGGCATAATACATAACGCCAAAAGCGAA	Top Polymerization Staples	Yellow
5[196]	4[196]	CCAAATTAGTAAATGAATTTTGTCGTCTTTCCAGACGAGGAG	Top Polymerization Staples	Yellow
3[189]	2[189]	CGACACCCAATAGGAACCCATTTTCAGGGATAGCAAGATGAC	Top Polymerization Staples	Yellow
25[196]	22[196]	CGCCATCGCGTCTGGCCTTCCCGCCATCAAAAATAATCCCTC	Top Polymerization Staples	Yellow
1[189]	0[189]	GCAGCAGAGGGTTGATATAAGCGGATAAGTGCCGTCGGAAAG	Top Polymerization Staples	Yellow
7[196]	6[196]	GGATTAGGAGCCTTTAATTGTAAAAAAAGGCTCCAAATTGCT	Top Polymerization Staples	Yellow
21[189]	18[189]	TACGTCCAATACTGCGGAATCTTAGACTGGATAGCGTAATGC	Top Polymerization Staples	Yellow
23[196]	20[196]	TGTAGGAAAAGCCCCAAAAACCCCCGGTTGATAATCACATTC	Top Polymerization Staples	Yellow
**Nano-hinge to Bottom Nano-brick Polymerzation Staples**				
Start	End	Sequence	Type	Color
11[21]	10[21]	AATCGGTGTATCACCGTACTCGTATAGCCCGGAATAGCAAGA	Bottom Polymerization Staples	Purple
13[21]	12[21]	GAAGTGTTTCGTCACCAGTACTGTACCGTAACACTGAATTAG	Bottom Polymerization Staples	Purple
15[28]	14[28]	ACGTCCTAAACAACTTTCAACTCTGTATGGGATTTTGACCAA	Bottom Polymerization Staples	Purple
17[28]	16[28]	AACGGTGCTTTCGAGGTGAATTATCGGTTTATCAGCTTAATC	Bottom Polymerization Staples	Purple
19[28]	18[28]	AAAAGCGTCACCCTCAGCAGCGCCGCTTTTGCGGGATGGTGA	Bottom Polymerization Staples	Purple
20[34]	21[34]	CCAACGGTTGTACCAAAAACAAGCATAAAGCTAAATCCTAAA	Bottom Polymerization Staples	Purple
22[34]	23[34]	AGGCGTAACCTGTTTAGCTATTTTCGCAAATGGTCAACAGAC	Bottom Polymerization Staples	Purple
24[34]	25[34]	CATTCCTGTAGCTCAACATGTATTGCTGAATATAATGAGTGA	Bottom Polymerization Staples	Purple
26[34]	27[34]	TCTACCGCGTTTTAATTCGAGGAAAGACTTCAAATATGTTAA	Bottom Polymerization Staples	Purple
31[21]	30[21]	ATAAATGAATCCCCCTCAAATCGTCATAAATATTCATTAAGG	Bottom Polymerization Staples	Purple
33[21]	32[21]	AGCCTAATATCGCGTTTTAATAGCCCGAAAGACTTCATTACA	Bottom Polymerization Staples	Purple
35[21]	34[21]	TTTATTGAATATAATGCTGTACTTAGAGCTTAATTGCCAAAA	Bottom Polymerization Staples	Purple
37[21]	36[21]	AGGAACAAATGGTCAATAACCCATTAGATACATTTCGTTGAG	Bottom Polymerization Staples	Purple
39[28]	38[28]	CGCTGAAATTAAGCAATAAAGAGGCAAAGAATTAGCAAGAGC	Bottom Polymerization Staples	Purple
41[28]	40[28]	TTAAAAATGCCTGAGTAATGTCATATATTTTAAATGCTCAGC	Bottom Polymerization Staples	Purple
63[21]	62[21]	AACCCTAACGTGCTTTCCTCGCTTTGACGAGCACGTATCGTT	Bottom Polymerization Staples	Purple
65[21]	64[21]	TCAGTCACGCAAATTAACCGTAAAAGAGTCTGTCCATTGAGA	Bottom Polymerization Staples	Purple
