# Supplementary material for: Real-time magnetic actuation of DNA nanodevices via modular integration with stiff micro-levers
Source: Nat Commun. 2018 Apr 13;9:1446. doi: 10.1038/s41467-018-03601-5 (PMC5899095; doi:10.1038/s41467-018-03601-5)
Supplement: Supplementary file 2 — Description of Additional Supplementary Files(PDF 177 kb) [file 41467_2018_3601_MOESM2_ESM.pdf]

Description of Additional Supplementary Files

File Name: Supplementary Movie 1
Description: Total internal reflection fluorescence (TIRF) video showing the thermal fluctuations of the lever arm polymerized from the top nano-brick labeled with Alexa 488, imaged at 5 fps.

File Name: Supplementary Movie 2
Description: TIRF video of actuation of lever arm labeled with Alexa 488 tethered to the surface and attached to 1 μm magnetic bead. Lever is actuated at frequencies of 1, 2 and 3 Hz by rotating an in-plane magnetic field of 40 Oe.

File Name: Supplementary Movie 3
Description: TIRF video of actuation of lever arms with multiple constructs in the field of view. Two lever arms are labeled with a magnetic bead, and one is not. The levers are actuated at a frequency of 0.5 Hz by rotating an in-plane magnetic field of 40 Oe.

File Name: Supplementary Movie 4
Description: TIRF video showing montage of two examples of directly manipulating the lever arm into multiple orientations, specifically four orthogonal orientations. The left video shows a sequential positioning into each orientation and the right video shows random selection of orientations. The position for manipulation is indicated by the white arrows.

File Name: Supplementary Movie 5
Description: TIRF video of actuation of polymerized nano-rotor where the nano-platform is fixed to the surface and the lever arms attached to the nano-rotor arm is labeled with Alexa 488 and attached on one end to a 1 μm magnetic bead. The rotor assembly is precessed at 1 Hz using an in-plane precessing magnetic field of 40 Oe.

File Name: Supplementary Movie 6
Description: TIRF video of actuation of polymerized nano-rotor where the nano-platform is fixed to the surface and the lever arms attached to the nano-rotor arm is labeled with Alexa 488 and attached on one end to a 1 μm magnetic bead. The rotor assembly is precessed at 1 Hz using an in-plane precessing magnetic field of 40 Oe.

File Name: Supplementary Movie 7
Description: TIRF video of actuation of polymerized nano-rotor where the nano-platform is fixed to the surface and the lever arms attached to a micro-lever labeled with Alexa 488 and attached on one end to a 1 μm magnetic bead. The rotor assembly is precessed at 2 Hz using an in-plane precessing magnetic field of 40 Oe. The rotor does not continuously track with the precessing field sometimes stalling and catching the field on a subsequent rotation.

File Name: Supplementary Movie 8
Description: TIRF video of actuation of a polymerized nano-hinge with bottom arm fixed to surface and top arm attached to 1 µm magnetic bead. The hinge assembly was open and closed using a precessing in-plane field of 40 Oe that repeats a sequence of rotating at a frequency of 1 Hz for one full rotation counter clockwise and then clockwise.

File Name: Supplementary Movie 9
Description: TIRF video of actuation of a polymerized nano-hinge with bottom arm fixed to surface and top arm attached to 1 µm magnetic bead. The hinge assembly was opened and closed by rotating an in-plane field of 40 Oe manually.

File Name: Supplementary Movie 10
Description: TIRF video illustrating the ability to hold the hinge in fixed configurations including an open configuration, closed configuration, and intermediate angle.

File Name: Supplementary Movie 11
Description: TIRF video showing two rotor assemblies actuated simultaneously. The left rotor only contains a single lever arm.
